# Bevacizumab increases the sensitivity of olaparib to homologous recombination-proficient ovarian cancer by suppressing CRY1 via PI3K/AKT pathway

**DOI:** 10.3389/fonc.2024.1302850

**Published:** 2024-02-14

**Authors:** Yasushi Iida, Nozomu Yanaihara, Yuki Yoshino, Misato Saito, Ryosuke Saito, Junya Tabata, Ayako Kawabata, Masataka Takenaka, Natsuko Chiba, Aikou Okamoto

**Affiliations:** ^1^ Department of Obstetrics and Gynecology, The Jikei University School of Medicine, Tokyo, Japan; ^2^ Department of Cancer Biology, Institute of Development, Aging and Cancer, Tohoku University, Sendai, Japan

**Keywords:** ovarian cancer, olaparib, bevacizumab, homologous recombinant proficient, CRY1

## Abstract

PARP inhibitors have changed the management of advanced high-grade epithelial ovarian cancer (EOC), especially homologous recombinant (HR)-deficient advanced high-grade EOC. However, the effect of PARP inhibitors on HR-proficient (HRP) EOC is limited. Thus, new therapeutic strategy for HRP EOC is desired. In recent clinical study, the combination of PARP inhibitors with anti-angiogenic agents improved therapeutic efficacy, even in HRP cases. These data suggested that anti-angiogenic agents might potentiate the response to PARP inhibitors in EOC cells. Here, we demonstrated that anti-angiogenic agents, bevacizumab and cediranib, increased the sensitivity of olaparib in HRP EOC cells by suppressing HR activity. Most of the γ-H2AX foci were co-localized with RAD51 foci in control cells. However, most of the RAD51 were decreased in the bevacizumab-treated cells. RNA sequencing showed that bevacizumab decreased the expression of CRY1 under DNA damage stress. CRY1 is one of the transcriptional coregulators associated with circadian rhythm and has recently been reported to regulate the expression of genes required for HR in cancer cells. We found that the anti-angiogenic agents suppressed the increase of CRY1 expression by inhibiting VEGF/VEGFR/PI3K pathway. The suppression of CRY1 expression resulted in decrease of HR activity. In addition, CRY1 inhibition also sensitized EOC cells to olaparib. These data suggested that anti-angiogenic agents and CRY1 inhibitors will be the promising candidate in the combination therapy with PARP inhibitors in HR-proficient EOC.

## Introduction

Poly-(ADP-ribose) polymerase inhibitors (PARPi) are orally active anticancer drugs causing synthetic lethality in cells with defects in homologous recombination (HR) DNA repair. PARP1 catalyzes the synthesis of poly-(ADP-ribose) (PAR) and transfers PAR to its substrates to enhance DNA single-strand break (SSB) repair (1). PARPi inhibit the catalytic activity of PARP1, resulting in delayed SSB repair, and trap PARP1 on SSBs, inducing DNA double-strand breaks (DSBs) and stalls of replication folks. The DSBs induced by PARPi would normally be repaired by HR. However, in cancer cells with HR deficient (HRD), the use of lower fidelity forms of DNA repair, such as non-homologous end-joining, significantly increases genomic instability, making repair unsustainable after multiple replications and resulting in tumor cell death (2).

The introduction of PARPi in clinical practice has greatly changed the management of patients with advanced high-grade epithelial ovarian cancer (EOC) in both first-line therapy and recurrent settings (3–8). Although PARPi are highly effective in treating EOC with HRD initially, virtually all patients develop resistance during over time (9). Additionally, EOC without HRD has primary resistance to PARPi and does not benefit from PARPi. Thus, a new strategy to overcome the resistance to PARPi is required.

A combination of PARPi and various chemotherapeutics or molecularly targeted agents has been developed to overcome the resistance to PARPi. Combinations of PARPi and chemotherapy, antiangiogenic agents, immune checkpoint inhibitors, tyrosine kinase inhibitors, and other inhibitors of DNA damage response are currently under investigation (10, 11). In particular, the combination of PARPi and antiangiogenic agents, including bevacizumab, has been extensively investigated. The phase III PAOLA-1 study of maintenance olaparib and bevacizumab in patients with newly diagnosed EOC demonstrated a substantial clinical benefit primarily in patients with HRD tumors (12). This led FDA approval of maintenance olaparib and bevacizumab only for EOC with HRD. Recent phase II clinical trials showed that the combination of PARPi and the antiangiogenic agent significantly improved progression-free survival (PFS) in patients with platinum-sensitive recurrent high-grade EOC compared with PARPi alone (13, 14). Interestingly, subgroup analyses of these trials showed that the improvement of PFS by the addition of antiangiogenic agents was independent of the HR status. These results showed that the combination of antiangiogenic agents and PARPi not only improves therapeutic efficiency in cancers with HRD but also sensitizes cancers without overt HRD to PARPi. However, the molecular mechanism of the improved therapeutic efficacy is unknown.

Antiangiogenic agents include antibodies against vascular endothelial growth factor (VEGF) or its receptor (VEGFR) and small-molecule inhibitors of VEGFR tyrosine kinase. These agents exert anticancer activity indirectly through the alteration in the endothelial function and directly by inhibiting the proliferation of signaling from VEGFR in cancer cells (15). Angiogenesis is essential for solid tumor growth and metastasis (16). VEGF and VEGFR are expressed at varying levels in EOC cells. Bevacizumab, a monoclonal antibody targeting VEGF-A, and cediranib, a small-molecule inhibitor targeting multiple factors, including VEGFRs 1–3 and c-kit, have demonstrated the antitumor activity in patients with EOC (17–20). At the time of this study, little is known about the role of the VEGF/VEGFR signaling pathway in HR. Recently, some genes and chemical agents that have not been considered to directly act on HR have been reported to affect HR activity. For example, inhibition of TTK protein kinase, which plays an important role in regulating spindle assembly checkpoint signaling, impaired HR in basal-like breast cancer cells (21), and a chemothrerapeutic agent paclitaxel, which exert its cytotoxic effect by arresting mitosis through microtubule stabilization, decease HR activity in HR-proficient (HRP) EOC cells (22). Thus, antiangiogenic agents may affect HR activity in EOC cells, which improves the sensitivity of these cells to PARPi.

This study aimed to investigate the molecular mechanism of the improvement of the antiproliferative effect by the combination of PARPi and antiangiogenic agents in EOC cell lines.

## Materials and methods

### Cell lines and reagents

OVSAHO, a high-grade serous ovarian cancer cell line, OVISE and OVTOKO, clear cell ovarian cancer (CCOC) cell lines, and TOV112D, an endometrioid ovarian cancer cell line, were purchased from the Japanese Collection of Research Bioresources Cell Bank, Osaka, Japan. Information on each cell line was obtained from DepMap Portal (https://depmap.org/portal/) and cBioPortal (https://www.cbioportal.org) in shown in **Supplementary Table S1**. Cells were maintained at 37°C in a humidified atmosphere of 5% CO_2_ in RPMI-1640 medium (Sigma-Aldrich, St. Louis, MO, USA) with 10% fetal bovine serum. Bevacizumab, olaparib, and cediranib were purchased from Selleck Biotech, Houston, TX, USA. KS-15, a small-molecule inhibitor of cryptochrome circadian regulator 1 (CRY1), was purchased from MedChemExpress, Monmouth Junction, NJ, USA.

### Cell viability assay

Cells were seeded in 96-well plates, incubated for 24 h, and treated with serially diluted olaparib with or without bevacizumab (20 µg/ml) or cediranib (5 μM) or KS-15 (20 µM). Cell viability was assessed after 6 days using the MTS assay. The MTS assay was performed using the CellTiter 96 AQueous One Solution Cell Proliferation Assay kit (Promega, Madison, WI, USA) according to the manufacturer’s instructions. Briefly, MTS solution was added to each of the 96-well plates and incubated for 1 h. Then, absorbance was measured at 490 nm using a microplate reader. Viability curves and the IC50 (half maximal inhibitory concentration) of each compound were calculated using GraphPad Prism 9 software (GraphPad Inc., San Diego, CA, USA). Reproducibility was confirmed by four independent experiments.

### Cell proliferation assay

Cell proliferation assay was performed with olaparib (50 µM) with or without bevacizumab (20 µg/ml) or cediranib (5 μM) or KS-15 (20 μM). The cell proliferation of olaparib alone was used as a control and was compared to that of the addition of bevacizumab or cediranib or KS-15, respectively. The experiment was repeated four times.

### Transfection

For the transfection of small interfering RNA (siRNA) alone and co-transfection of siRNA and plasmid, the Trans-IT X2 dynamic delivery system (Mirus BIO, Madison, WI, USA) was used according to the manufacturer’s instructions. A predesigned siRNA targeting *CRY1* (Silencer Select Predesigned siRNA, Assay ID: s464) and a non-targeted control siRNA (Silencer Select Negative Control No. 1) were purchased from Thermo Fisher Scientific (Waltham, MA, USA).

### HR activity assay

HR activity was analyzed using the Assay for Site-specific HR Activity (ASHRA) (23). Cells were seeded in 6-well plates, incubated for 24 h, and treated with bevacizumab (20 µg/ml), cediranib (5 μM), and siRNA targeting *CRY1*. The donor vector (Addgene ID: #169798), the expression vector for gRNA, and Cas9 (Addgene ID: #169795 and #169796) were transfected using Transporter 5 Transfection Reagent (Polysciences, Warrington, PA, USA) according to the manufacturer’s instructions. After 48 h incubation, genomic DNA was extracted, and quantitative polymerase chain reaction (PCR) was performed on the StepOnePlus real-time PCR System (Applied Biosystems, Foster City, CA, USA) using Go Taq Green Master Mix (Promega). The knocked-in and control alleles were amplified with the following primer sets: 5’-GTCCTGCTGGAGTTCGTGACCG-3’ and 5’-GTGCAATCAAAGTCCTCGGC-3’ for the knocked-in allele and 5’-AGTTGCGTTACACCCTTTCTTG-3’ and 5’-GTGCAATCAAAGTCCTCGGC-3’ for the control allele. The relative quantity of the knocked-in allele was calculated using the 2^−ΔΔ CT^ method. Data were collected as the average values of each group and presented as mean ± standard deviation (SD). Each experiment was repeated at least three times.

### Immunofluorescence staining

OVISE cells were seeded in 8-well chambered slides at a density of 5.5 × 10^3^ cells per well, incubated for 24 h, and then treated with bevacizumab. Cells were irradiated with 2 Gy X-ray and fixed by chilled methanol 2 h after irradiation. After permeabilization by 1% TritonX-100 and blocking by a blocking solution in DNA Damage Detection Kit-gH2AX, the samples were incubated with primary antibodies diluted in a blocking solution in DNA Damage Detection Kit-gH2AX at 4°C overnight. Then, the samples were incubated with secondary antibodies diluted in a blocking solution in a DNA Damage Detection Kit-gH2AX at room temperature for 1 h with 4′,6-diamidino-2-phenylindole (DAPI, Dojindo, Kumamoto, Japan) and mounted in Vectashield. Images were observed under a fluorescence microscope (BZ-X800, Keyence, Osaka, Japan). Antibodies, including anti-RAD51 (14961-1-AP; 1:200, Proteintech, Rosemont, IL, USA), anti-γ-H2AX (in DNA Damage Detection Kit-gH2AX-Green, G265, Dojindo), and goat anti-rabbit IgG conjugated with Texas Red (4050-07; 1:200, SouthernBiotech, Birmingham, AL, USA), were used. A total of 30 cells from three random fields per sample were observed to quantify the RAD51 and gH2AX foci formation. Cells with more than five foci were considered positive, and the fraction of foci-positive cells was calculated. The average ratio of RAD51-positive cells/gH2AX-positive cells in each sample was presented with SD. Each experiment was repeated at least two times.

### RNA sequencing

OVISE cells were seeded in 6 cm dishes and incubated for 24 h. Cells were treated with or without bevacizumab (20 µg/ml) and incubated for 192 h. Total RNA was extracted from these cells 2 h after treatment with 2 Gy of γ-irradiation. RNA sequencing was performed at the Kazusa DNA Research Institute. The data discussed in this study have been deposited in the NCBI’s Gene Expression Omnibus and are accessible through GEO Series accession number GSE203044. Purified total RNA was used for RNA library preparation according to the instructions of the Quant Seq 3’ mRNA-seq library preparation kit FWD for Illumina (Lexogen, Vienna, Austria). Libraries were sequenced using single-end 75 bp on a NextSeq500 instrument to an average depth of 2.8 M clusters per sample. All data analyses were performed using Strand NGS 3.4 (Strand Life Sciences Pvt. Ltd., Bengaluru, India). In addition to trimming adapters and poly-A from FASTQ files, all read sequences were trimmed by 6 bp from the 5′ ends according to the manufacturer’s instructions. Reads were mapped to the human genome hg19. After DESeq normalization, a gene was considered differentially expressed if the adjusted *P*-value was <0.05 and fold-change >2 or <0.5. A Volcano plot was created using GEO2R (https://www.ncbi.nlm.nih.gov/goe/geo2r/). For enrichment analysis of the differentially expressed genes, gene set enrichment analysis was used to perform Gene Ontology analysis.

### Quantitative real-time reverse transcription-PCR analysis

RT-PCR analysis was performed as described previously (24). Briefly, extracted RNAs were subjected to RT using qScript cDNA SuperMix (Quantabio, Beverly, MA, USA), followed by quantitative real-time RT-PCR using TaqMan Fast Advanced Master Mix (Thermo Fisher Scientific). All PCR reactions were performed in 96-well plates using the StepOnePlus real-time PCR System (Applied Biosystems). Glyceraldehyde 3-phosphate dehydrogenase was used as an endogenous control, and untreated cells were set as the reference. Gene expressions were quantified using the comparative 2^−ΔΔCT^ method.

### Western blotting

Western blotting analysis was performed as described previously (24). Cells were collected 48 hours after administration of LY294002 and bevacizumab and transfection with siRNA VEFR2. Irradiation and administration of olaparib were performed 2 and 8 hours before cell collection, respectively. Total protein was resolved on gradient NuPage 4%–12% Bis-Tris gels (Thermo Fisher Scientific) and transferred to membranes using an iBlot1 Gel Transfer Device (Thermo Fisher Scientific). The membranes were incubated sequentially with primary antibodies at 4°C and horseradish peroxidase-conjugated secondary anti-rabbit or anti-mouse antibody (1:10000, Cell Signaling Technology, Beverly, MA) at room temperature with gentle agitation. Positive immunoreactions were detected using the ImmunoStar LD chemiluminescence system (Wako, Tokyo, Japan). Rabbit polyclonal antibody against CRY1 (EPR165; #ab229631; 1:1000) was purchased from Abcam (Cambridge, UK), and rabbit monoclonal antibodies against AKT (11E7; #4685; 1:1000), phosphorylated AKT (Thr308) (244F9; #4056; 1:1000), phosphorylated AKT (Ser473) (D9E; #4060; 1:2000) and β-actin (13E5; #4970; 1:4000) were purchased from Cell Signaling Technology.

### Enzyme-linked immunosorbent assays

ELISAs were performed as described previously (25). Briefly, cells were seeded in 6 cm dishes and incubated for 72 h. Part of the culture supernatant was collected immediately in the control group, 2 h after irradiation in the irradiation group, 8 h after olaparib administration in the olaparib group and 48 h after bevacizumab administration and 2 h after irradiation in the irradiation and bevacizumab group, respectively. Four samples from each group were collected. ELISA analysis of VEGF concentration was performed using DVE00 for VEGF ELISA kits (R&D Systems, Minneapolis, MN, USA). The mean concentration of VEGF was compared between the control group and the irradiation and olaparib groups.

### Statistical analysis

All statistical analyses were performed using GraphPad Prism 9 software (GraphPad Inc.). Means of the control and experimental groups were compared using one-way or two-way analysis of variance, followed by Tukey’s multiple comparison tests. Statistical significance was set at *P* < 0.05.

### Data availability

All data generated or analysed during this study are included in this published article (and its **Supplementary Information Files**). The datasets generated and analysed during the current study are available in the NCBI’s Gene Expression Omnibus repository, accession number GSE203044.

## Results

### Combination with antiangiogenic agents enhances the effect of olaparib

**Figure 1:**
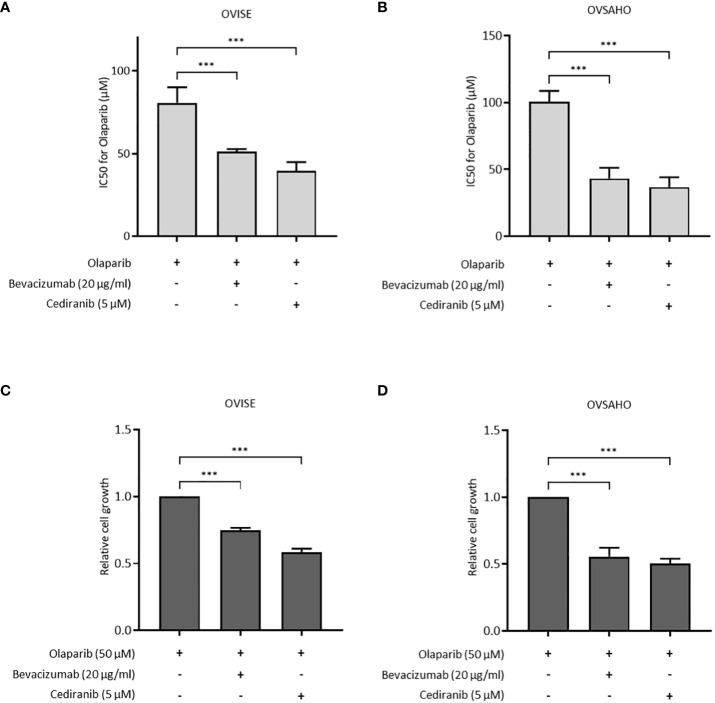
The effect of olaparib was enhanced by the addition of antiangiogenesis in homologous recombination-proficient (HRP) cells. Data are shown as mean ± standard deviation (SD). ***P < 0.001. **(A, B)** IC50 (half maximal inhibitory concentration) values based on the viability of OVISE and OVSAHO cells treated with olaparib with or without antiangiogenesis. **(C, D)** Growth inhibition of OVISE and OVSAHO cells treated with olaparib with or without antiangiogenesis.

The antiproliferative effect of olaparib with and without antiangiogenic agents was assessed in OVISE and OVSAHO cells. OVISE cells have no alteration in genes associated with HR, except for amplifications of unknown biological effect in the *BRCA1* and *NBN* genes, and are considered HRP. OVSAHO cells have deletions in the *BRCA2* and *CHEK1* genes that are considered to be oncogenic and are considered HRD. In OVISE cells, the IC50 of olaparib alone was 80.73 μM, which was significantly higher than that under co-treatment with bevacizumab or cediranib (51.18 μM or 39.55 μM, respectively) (**Figure 1A**, **Supplementary Figure S1A**). Similar results were seen in OVSAHO cells (**Figure 1B**, **Supplementary Figure S1B**). Co-treatment with bevacizumab or cediranib inhibited cell proliferation more than olaparib alone in both OVISE and OVSAHO cells (**Figures 1C, D**).

### Inhibition of the VEGF signaling pathway suppressed HR activity through the downregulation of *CRY1* expression

**Figure 2:**
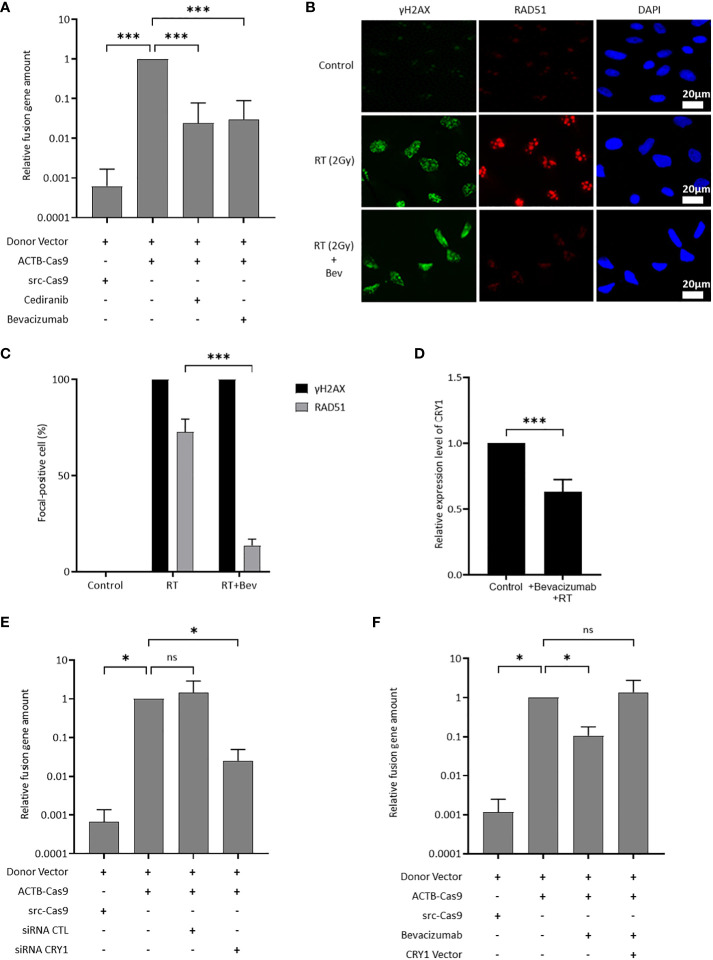
*CRY1* is involved in the reduction of HR activity by the addition of antiangiogenesis. Data are shown as mean ± SD. ***P < 0.001; *P < 0.05; ns, not significant. **(A)** HR activity in OVISE cells treated with cediranib and bevacizumab was analyzed by the Assay for Site-specific HR Activity (ASHRA). **(B)** Representative images of immunofluorescence staining showing the RAD51 foci in different groups for OVISE cells. γH2AX, RAD51, and DAPI are shown in green, red, and blue, respectively. Radiation increased the expression of γH2AX and RAD51, and bevacizumab suppressed the expression of RAD51 increased by radiation. **(C)** Data were collected as the average ratio (RAD51 positive cells/gH2AX positive cells) of each group. **(D)** Relative expression levels of *CRY1* in OVISE after irradiation and the addition of bevacizumab were evaluated by reverse transcription polymerase chain reaction (RT-PCR). **(E)** HR activity in OVISE cells treated with siRNA *CRY1* was analyzed by ASHRA. **(F)** HR activity in OVISE cells treated with bevacizumab with or without *CRY1* vector was analyzed by ASHRA.

The effect of the inhibition of the VEGF pathway on HR activity was evaluated using ASHRA to elucidate the mechanism of sensitization to olaparib by the inhibition of the VEGF pathway. ASHRA can quantify cellular HR activity, and the measured activity in ASHRA correlates linearly with sensitivity to PARP inhibitors (26). The treatment with the addition of bevacizumab or cediranib significantly suppressed the HR activity in OVISE cells (**Figure 2A**). The intranuclear foci formation of RAD51, a marker of functional HR, was examined after X-ray irradiation to confirm the suppression of HR activity. Most of the γ-H2AX foci, a marker of DNA damage, were co-localized with RAD51 foci in control cells. However, most of the foci formation of RAD51 was decreased in the bevacizumab-treated cells (**Figures 2B, C**). These findings showed that the inhibition of the VEGF pathway suppressed HR activity in these cells.

Differentially expressed genes were investigated by RNA sequencing to identify a mediator of suppression of HR activity by the inhibition of the VEGF pathway. The results showed that bevacizumab treatment decreased the expression of *CRY1* in X-ray-irradiated OVISE cells (**Supplementary Figure 2A, B**, **Supplementary Tables S2**). The decreased *CRY1* expression was confirmed by real-time PCR analysis (**Figure 2D**). *CRY1* is a circadian gene that regulates the expression of several genes associated with HR. Thus, the HR activity in *CRY1*-knockdown cells was evaluated. The knockdown of *CRY1* by RNAi significantly suppressed the HR activity in OVISE cells (**Figure 2E**). Interestingly, the exogenous expression of *CRY1* rescued the suppression of HR activity in the bevacizumab-treated cells (**Figure 2F**).

### Bevacizumab suppressed CRY1 expression via PI3K/AKT pathway, and inhibition of CRY1 increased the effect of olaparib

**Figure 3:**
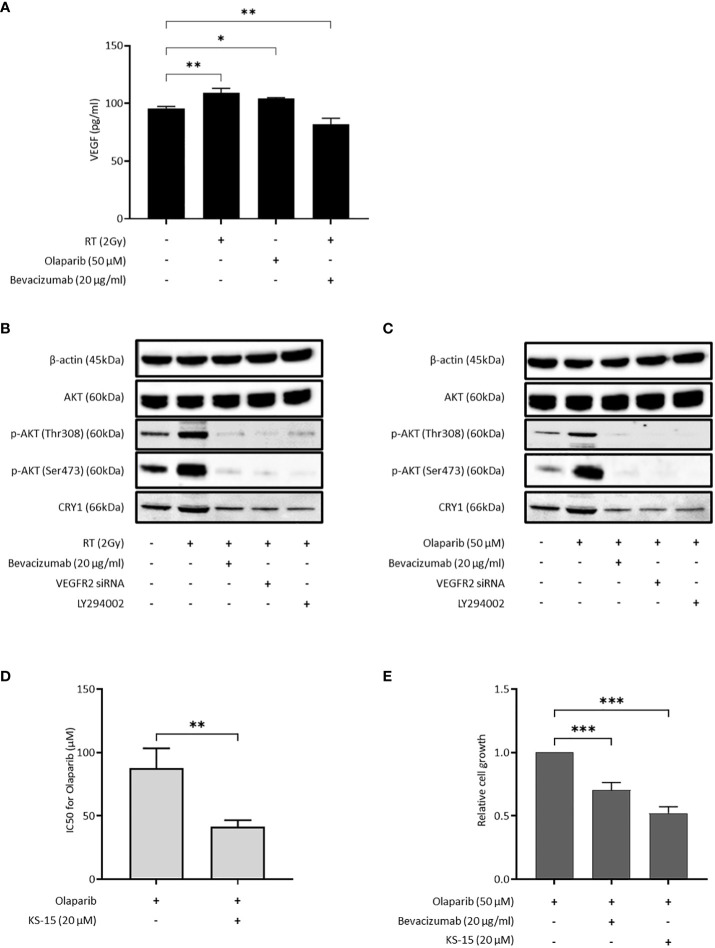
Bevacizumab suppresses CRY1 expression via the PI3K/AKT pathway. The inhibition of CRY1 enhanced the effect of olaparib in HRP cells. Data are shown as mean ± SD. ***P < 0.001; **P < 0.01; *P < 0.05. **(A)** VEGF released by OVISE in response to irradiation and olaparib were determined by enzyme-linked immunosorbent assay (ELISA). **(B)** CRY1 expression in OVISE cells was evaluated by Western blot when bevacizumab or VEGFR2 siRNA or LY294002 was added to irradiation. Cells were collected 2 hours after irradiation. **(C)** CRY1 expression in OVISE cells was evaluated by Western blot when bevacizumab or VEGFR2 siRNA or LY294002 was added to olaparib. **(D)** IC50 values based on the viability of OVISE cells treated with olaparib with or without KS-15, an inhibitor of CRY1. **(E)** Growth inhibition of OVISE cells treated with olaparib with or without KS-15.

Because both bevacizumab and cediranib suppressed HR activity, we speculated that VEGF might be produced in cancer cells under DNA damage stress. VEGF in the culture medium of OVISE cells was increased by X-ray irradiation and the olaparib treatment, and decreased by X-ray irradiation and bevacizumab treatment (**Figure 3A**). Consistent with this, the phosphorylated fraction of AKT was significantly increased by irradiation (**Figure 3B**). Additionally, the increase in the expression of CRY1 protein by irradiation was confirmed (**Figure 3B**). When X-ray-irradiated OVISE cells were treated with bevacizumab or transfected with siRNA against VEGFR2, CRY1 and the phosphorylation of AKT were significantly decreased (**Figure 3B**). CRY1 expression is regulated by the PI3K/AKT pathway via inhibition of the dimer formation of CLOCK and BMAL2, the upstream regulator of *CRY1* (27, 28). The cells were treated with a PI3K inhibitor LY293002 to investigate whether bevacizumab suppressed the expression of CRY1 via inhibition of the PI3K/AKT pathway and observed a similar decrease in CRY1 and the phosphorylation of AKT. The increase in CRY1 and the phosphorylation of AKT were also induced by olaparib treatment and again suppressed by the bevacizumab treatment, VEGFR2 knockdown, or PI3K inhibition (**Figure 3C**). Additionally, we confirmed that the olaparib treatment increased CRY1 and the phosphorylation of AKT, which was suppressed by the bevacizumab treatment, using another clear cell carcinoma cell line, OVTOKO. A similar phenomenon was observed in non-clear cell carcinoma cell lines, OVSAHO and TOV-112D (**Supplementary Figure S3**).

The cells were treated with KS-15, a CRY1 inhibitor, concomitantly with olaparib to elucidate the importance of CRY1 in the antiproliferative effect of olaparib. The co-treatment with KS-15 significantly decreased the IC50 of OVISE cells compared with olaparib alone (**Figure 3D**, **Supplementary Figure S4**). Furthermore, similar to the co-treatment with bevacizumab, the co-treatment with KS-15 suppressed cell proliferation more than olaparib alone (**Figure 3E**).

## Discussion

**Figure 4:**
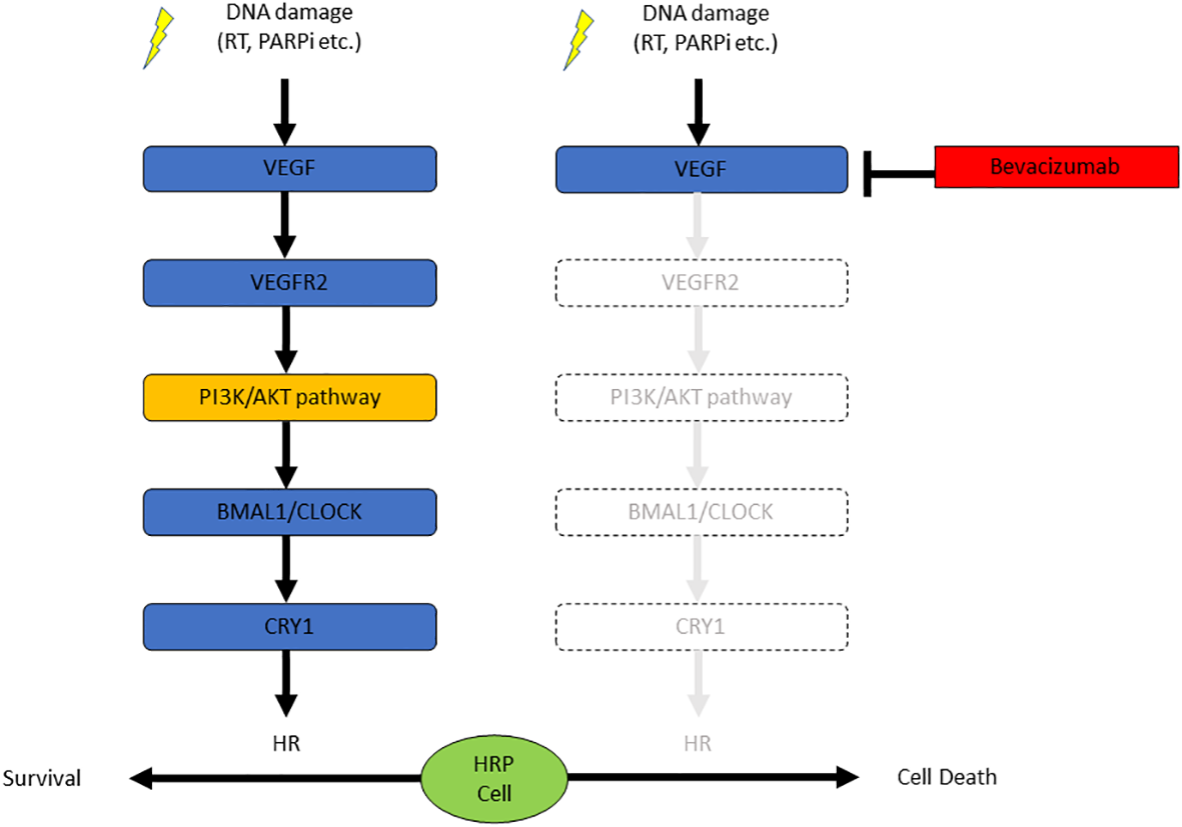
The combination of bevacizumab and olaparib is effective against HRP ovarian cancer cells due to the suppression of CRY1 via the PI3K/AKT pathway.

In this study, we found the blockade of VEGF/VEGFR signaling suppressed the HR activity in EOC cells without obvious mutations in HR-related genes, resulting in the sensitization of the HRP EOC cells to PARPi. DNA damage stress induced by X-ray irradiation or PARPi activated the VEGF/VEGFR signaling pathway, which increased the expression of *CRY1*. *CRY1* enhanced the HR activity. Thus, antiangiogenic agents may potentiate the therapeutic effect of PARPi via inhibition of the VEGFR-PI3K/AKT-CRY1 axis (**Figure 4**).

The VEGF/VEGFR signaling pathway, which is directory activated by a transcription factor hypoxia-inducible factor-1, is a well-known regulator of angiogenesis (29).

Bevacizumab or cediranib, antiangiogenic agents, inhibit the VEGF/VEGFR signaling in vascular endothelial cells (30). However, the VEGF/VEGFR signaling also directly regulates cell survival, proliferation, metastasis, and sensitivity to chemotherapeutics in cancer cells (31). VEGF is produced in various cells, including cancer cells or non-neoplastic stromal cells (31). This study showed that X-ray irradiation or PARPi treatment increased the VEGF concentration in the culture media (**Figure 3A**). Although the precise mechanism is unknown, these data indicate that DNA damage stress stimulated VEGF production in cancer cells. The increased VEGF activated the downstream PI3K/AKT pathway, which was shown by the increase in the phosphorylated AKT fraction (**Figures 3B, C**, **Supplementary Figure S3**). The activating mutations in *PIK3CA* are common in clear cell carcinoma and relatively rare in the other histological subtypes (32). Since the increase in phosphorylated AKT by DNA damage stress was observed not only in clear cell carcinoma cell lines but also in cell lines of other histological subtypes (**Figures 3B, C**, **Supplementary Figure S3**), the activation of VEGFR-PI3K/AKT axis might be independent of *PI3KCA* mutation.

*CRY1* is one of the transcriptional coregulators associated with circadian rhythm (33). Disturbance of the circadian rhythm has recently been identified as an independent risk for cancer and classified as a carcinogen (33). Furthermore, circadian rhythm affects several hallmark phenotypes of cancer, including alterations in cell proliferation, survival, DNA repair, and metabolic regulation (33). Recent research showed that DNA damage stabilized CRY1, and the stabilized CRY1 temporally regulated the expression of genes required for HR in cancer cells (34). This study reported an increase in the *CRY1* expression by DNA damage, including X-ray irradiation and PARPi treatment, and the knockdown of *CRY1* by RNAi significantly suppressed the HR activity (**Figures 2D–F**, **3B, C**, **Supplementary Figure S3**). These data support the contribution of CRY1 in the enhancement of DNA damage repair by HR. Additionally, bevacizumab decreased the *CRY1* expression in irradiated cells and suppressed the HR activity (**Figures 2A, D**) and the suppression of HR activity by bevacizumab was restored by the exogenous expression of *CRY1* (**Figure 2F**). Circadian rhythm is influenced by several factors such as light and temperature (35), and the possibility that the expression CRY1 was influenced by these factors cannot be ruled out. However, the results of this study showed that bevacizumab inhibited an increase in the *CRY1* expression induced by DNA damage, resulting in the suppression of HR activity and enhancement of the PARPi effect. Furthermore, the PI3K inhibition or the knockdown of VEGFR2, which blocks the upstream signaling of PI3K decreased the CRY1 expression under DNA damage stresses as bevacizumab did (**Figures 3B, C**). These data indicate that inhibition of the VEGFR-PI3K/AKT-CRY1 axis may be sufficient to suppress HR activation by the increase in *CRY1*. Several clinical trials reported that the combination of PARPi and PI3K/AKT inhibitors showed enhanced efficacy regardless of cancer type and HR status (36–38). These results are consistent with those of our study.

KS-15, an inhibitor of CRY1, increased the sensitivity of olaparib as bevacizumab did (**Figures 3D, E**). Interestingly, KS-15 has different effects depending on the cell type. KS-15 exerted an antiproliferative effect and increased sensitivity to doxorubicin in the breast cancer cell line MCF7 but not in the non-transformed mammary epithelial cell line MCF10A (39). KS-15 showed a protective effect in non-neoplastic cells against cisplatin by promoting DNA repair and arresting the cell cycle (40). These results indicate that KS-15 selectively potentiates the therapeutic anticancer effect agents in transformed cells. Future studies are needed to investigate the mechanism of selective potentiation of therapeutic agents by KS-15. Interestingly, the enhancement of growth inhibition combined with olaparib was sustained for a longer period by KS-15 compared with bevacizumab (**Supplementary Figure S5**). The suppression of *CRY1* by bevacizumab was attenuated as time went by X-ray-irradiated cells (data not shown). This may be due to the rhythmic nature of *CRY1* expression regulation. Thus, KS-15 may be a better agent to inhibit HR activation in olaparib-treated cells and is a good candidate worth testing in combination with olaparib in clinical trials.

Preclinical studies showed that antiangiogenic agents affect HRR through various mechanisms, indicating synergy between PARPi and antiangiogenic agents. By blocking angiogenesis, antiangiogenic agents induce hypoxia in the microenvironment, and the hypoxic conditions lead to decreased expression of *BRCA1/2* and *RAD51* (41–43). Furthermore, VEGFR3 inhibition downregulates *BRCA* genes, and cediranib directly represses *BRCA1/2* and *RAD51* gene expression (41, 44). In this study, the antiangiogenic agents, bevacizumab or cediranib, enhanced the effect of olaparib in HRP EOC cells through a mechanism that is not associated with hypoxia induced by antiangiogenic agents reported to date.

Although the underlying mechanisms of these combinations are still not fully understood, clinical trials have been conducted to evaluate the combination of PARPi and antiangiogenic agents. In two phase II studies on patients with platinum-sensitive recurrent EOC, the combination of PARPi and antiangiogenic agents significantly improved PFS compared with PARPi alone (13, 14). A phase III study on patients with recurrent platinum-sensitive EOC, which compared the combination of cediranib and olaparib or olaparib alone with standard platinum-based chemotherapy, demonstrated that the median PFS was 10.4, 8.2, and 10.3 months for the combination, olaparib alone, and chemotherapy, respectively, and the results were similar in patients without germline *BRCA* (g*BRCA*) mutation (45). Another phase II study on heavily pre-treated patients with platinum-resistant recurrent EOC, which compared the combination of olaparib and cediranib or olaparib alone with weekly paclitaxel, demonstrated that the median PFS was 5.7, 3.8, and 3.1 months for the combination, olaparib alone, and weekly paclitaxel, respectively, and no significant difference in PFS was observed between the combination and weekly paclitaxel, and in the subgroup analysis of patients with wild-type g*BRCA*, the median PFS was 5.8, 3.8, and 2.1 months for the combination, olaparib alone, and weekly paclitaxel, respectively, indicating that the combination therapy showed a promising trend toward improved PFS compared with weekly paclitaxel (46). These results indicate that the combination of PARPi and antiangiogenic agents prolongs PFS compared with PARPi alone, but its efficacy has not been shown to be superior to standard platinum-based chemotherapy regardless of the g*BRCA* mutation status. Therefore, the combination of PARPi and antiangiogenic agents may be a viable alternative to chemotherapy for patients with recurrent EOC, particularly platinum-resistant recurrent EOC patients with wild-type g*BRCA*. The combination of PARPi and antiangiogenic agents was first evaluated in the phase III PAOLA-1 study as a maintenance treatment in the first-line setting, which reported a statistically significant improvement in the median PFS for olaparib and bevacizumab compared with placebo and bevacizumab in the overall population, and in the subgroup analysis, a substantial PFS benefit was observed with the combination treatment compared with bevacizumab alone in the HRD population but not in the HRP population (12). The lack of an olaparib alone arm makes it difficult to determine whether the combination has synergistic effects.

In conclusion, VEGF/VEGFR/PI3K signaling enhanced HR activity through the increase in the expression of *CRY1*. The study findings indicate that the antiangiogenic agents and the CRY1 inhibitors are promising combination partners to overcome primary resistance to PARPi by turning HRP cells into HRD cells. Furthermore, antiangiogenic agents and CRY1 inhibitors may concur with the secondary resistance to PARPi due to the activation of HR. These data provide an important molecular basis for the development of new therapeutic strategies for EOC.

## Data availability statement

The datasets presented in this study can be found in online repositories. The names of the repository/repositories and accession number(s) can be found in the article/**Supplementary Material**.

## Ethics statement

Ethical approval was not required for the studies on humans in accordance with the local legislation and institutional requirements because only commercially available established cell lines were used. Ethical approval was not required for the studies on animals in accordance with the local legislation and institutional requirements because only commercially available established cell lines were used.

## Author contributions

YI: Conceptualization, Funding acquisition, Methodology, Resources, Writing – original draft. NY: Conceptualization, Funding acquisition, Methodology, Resources, Writing – original draft. YY: Methodology, Writing – original draft. MS: Methodology, Visualization, Writing – review & editing. RS: Methodology, Writing – review & editing. JT: Methodology, Writing – review & editing. AK: Methodology, Writing – review & editing. MT: Methodology, Writing – review & editing. NC: Conceptualization, Writing – review & editing. AO: Conceptualization, Supervision, Writing – review & editing.
