# Supplementary material for: Bevacizumab increases the sensitivity of olaparib to homologous recombination-proficient ovarian cancer by suppressing CRY1 via PI3K/AKT pathway
Source: Front Oncol. 2024 Feb 14;14:1302850. doi: 10.3389/fonc.2024.1302850 (PMC10899666; doi:10.3389/fonc.2024.1302850)
Supplement: Supplementary file 1 [file DataSheet_1.pdf]

A

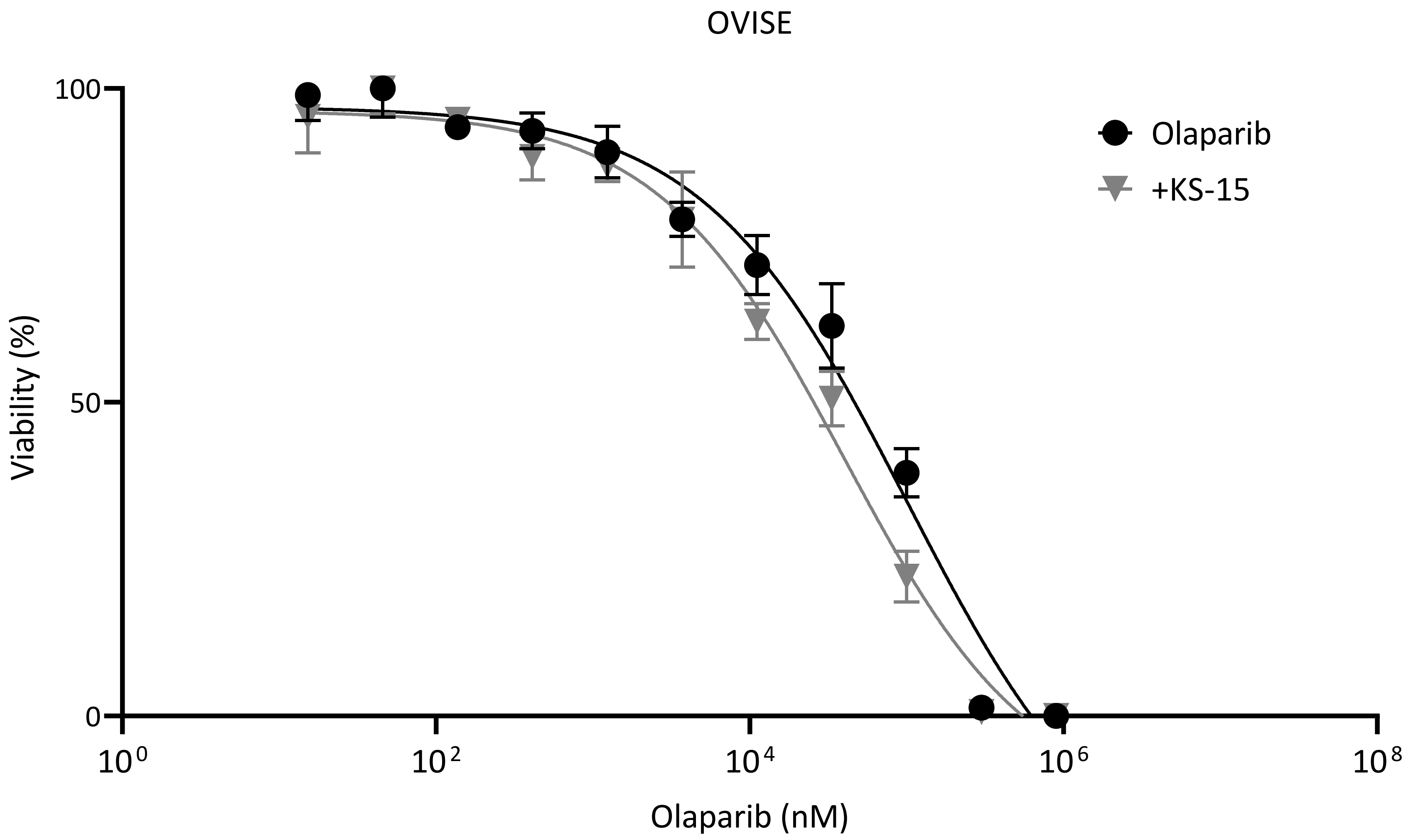

B

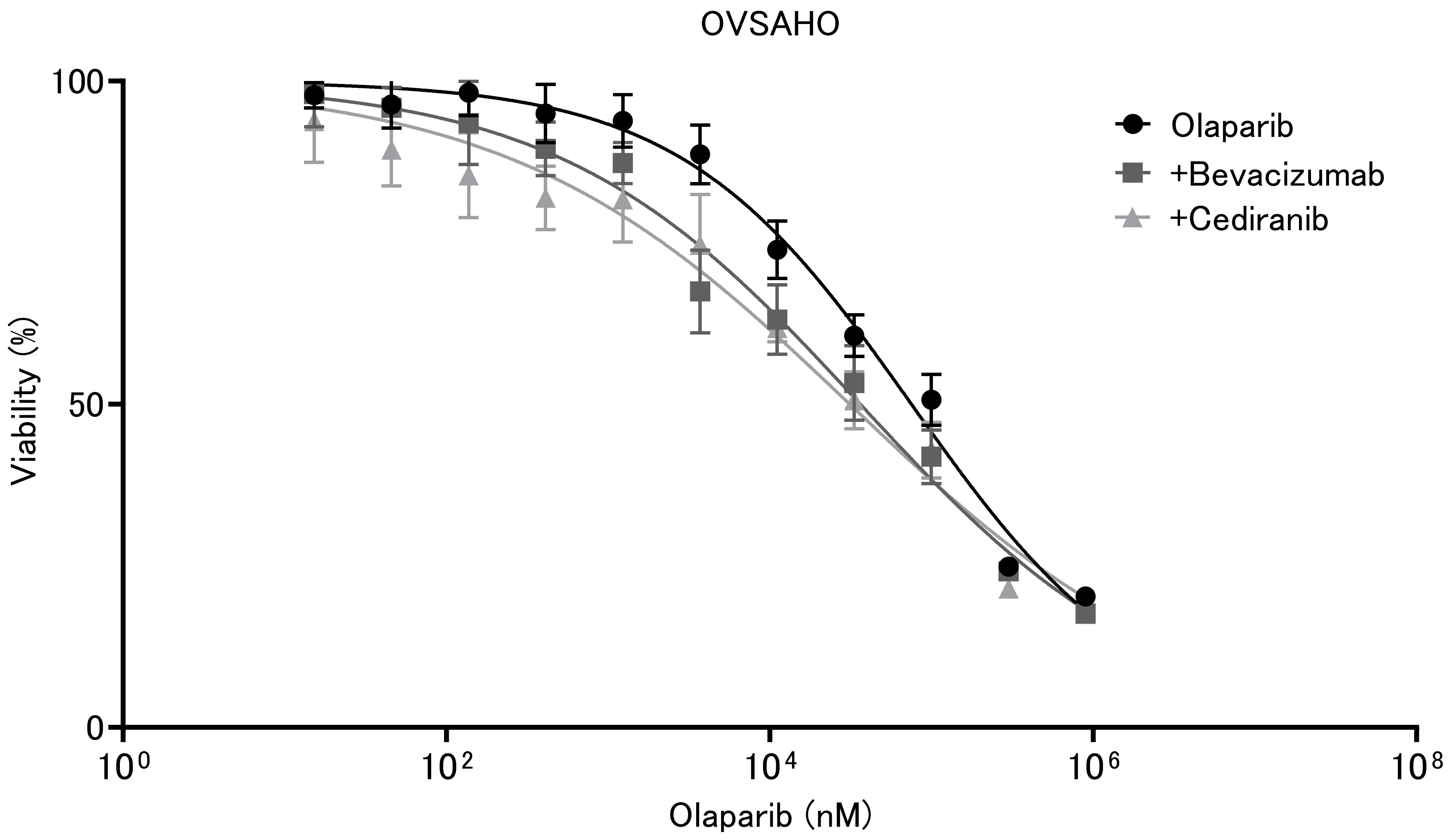

Supplementary Fig. S1. (A, B) Viability of OVISE and OVSAHO cells treated serially diluted olaparib with or without Bevacizumab (20 µg/ml) or cediranib (5 µM). Data are shown as mean ± standard deviation (SD).

A

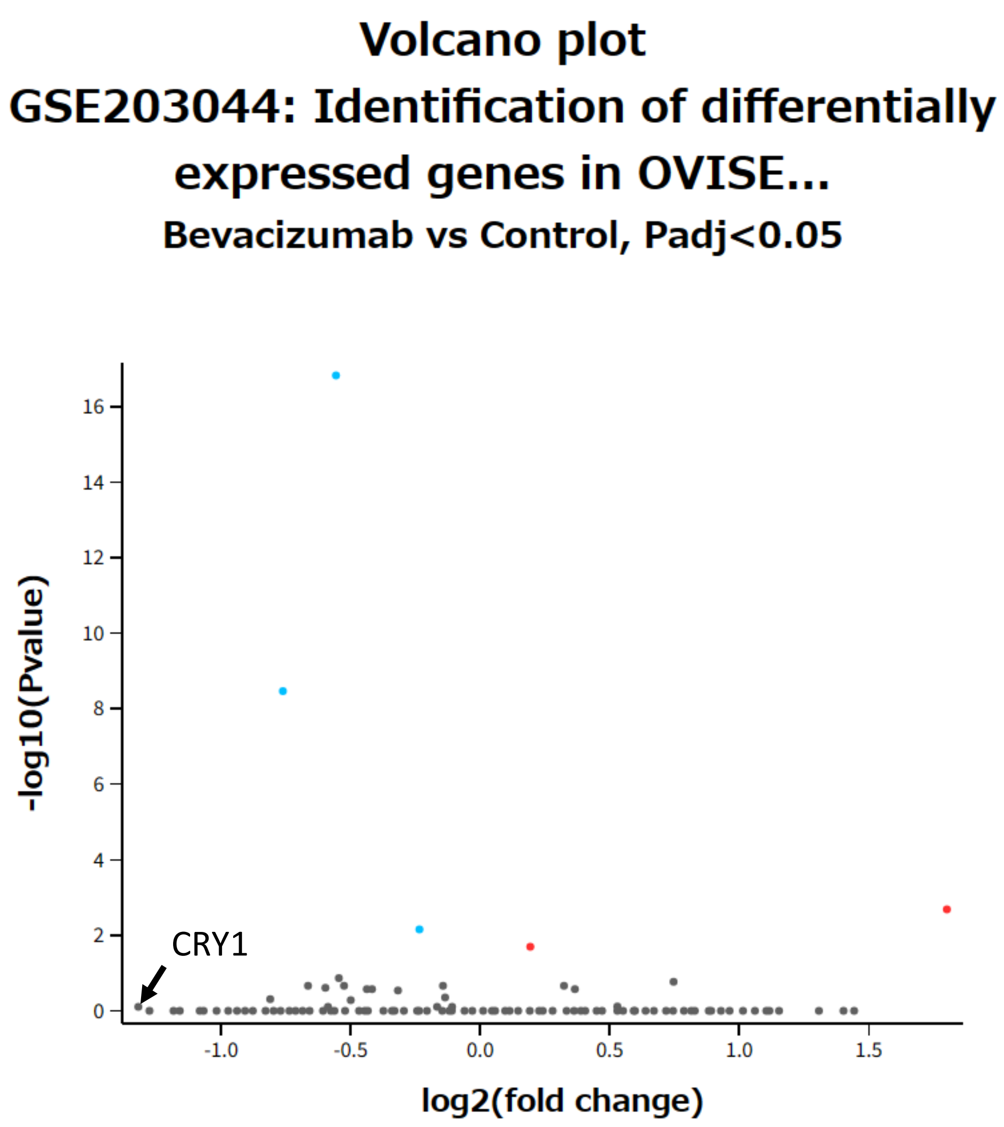

B

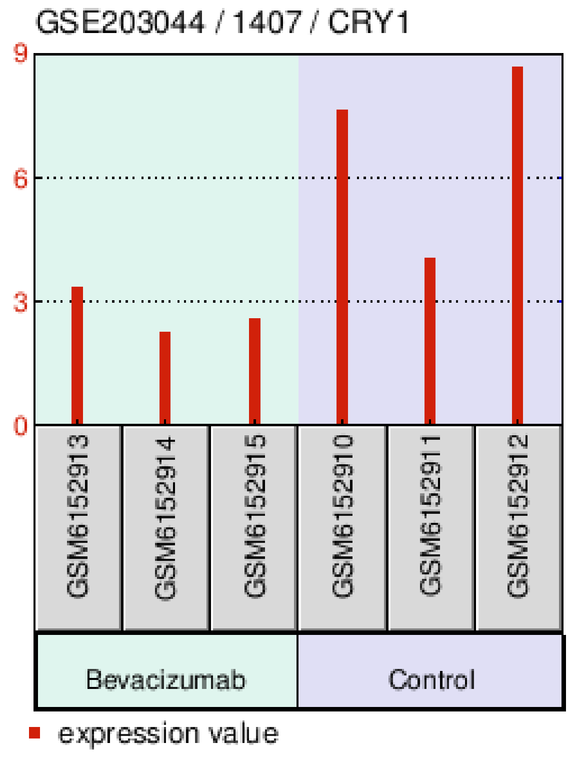

Supplementary Fig. S2. (A) Highlighted genes are significantly differentially expressed at p-value cutoff of 0.05 (red=upregulated, blue=downregulated) in Volcano plot. (B) The log2 fold change for bevacizumab compared to control was -1.318.

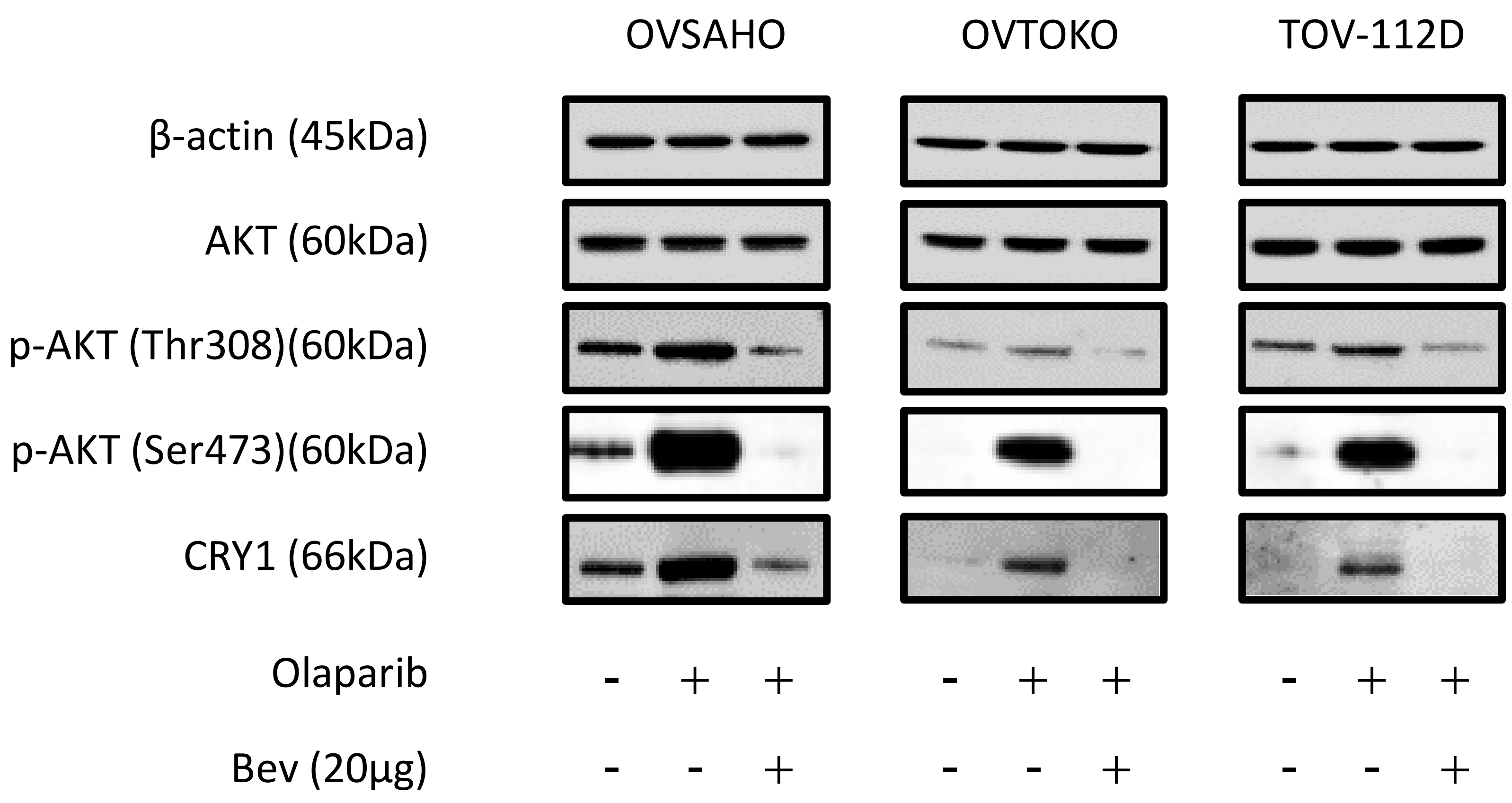

Supplementary Fig. S3. Bevacizumab suppressed *CRY1* expression via the PI3K/AKT pathway. *CRY1* expression in OVSAHO, OVTOKO, and TOV-112D cells was evaluated by Western blot when bevacizumab was added to olaparib.

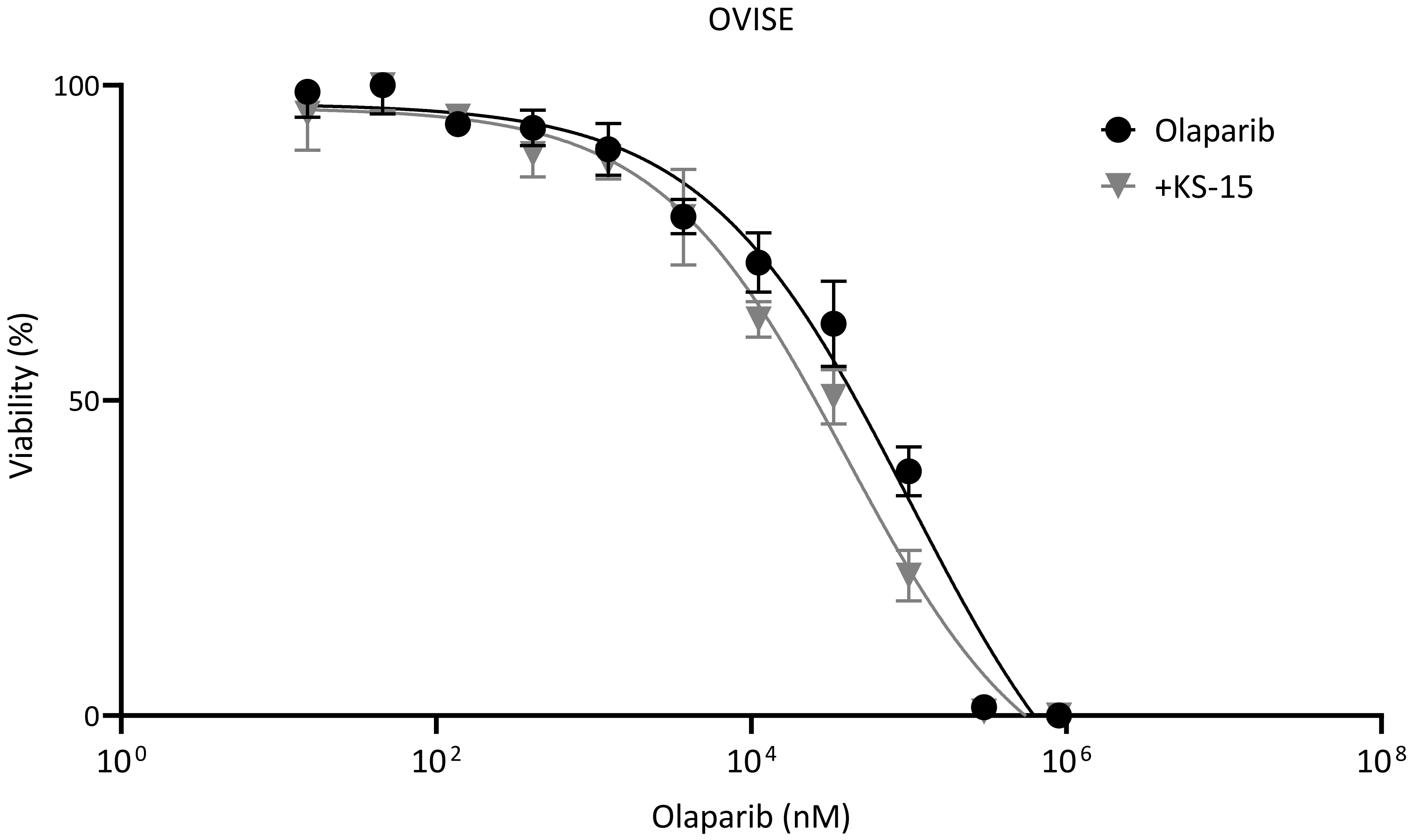

Supplementary Fig. S4. (A) Viability of OVISE cells treated with serially diluted olaparib with or without KS-15 (20 μM). Data are shown as mean ± standard deviation (SD).

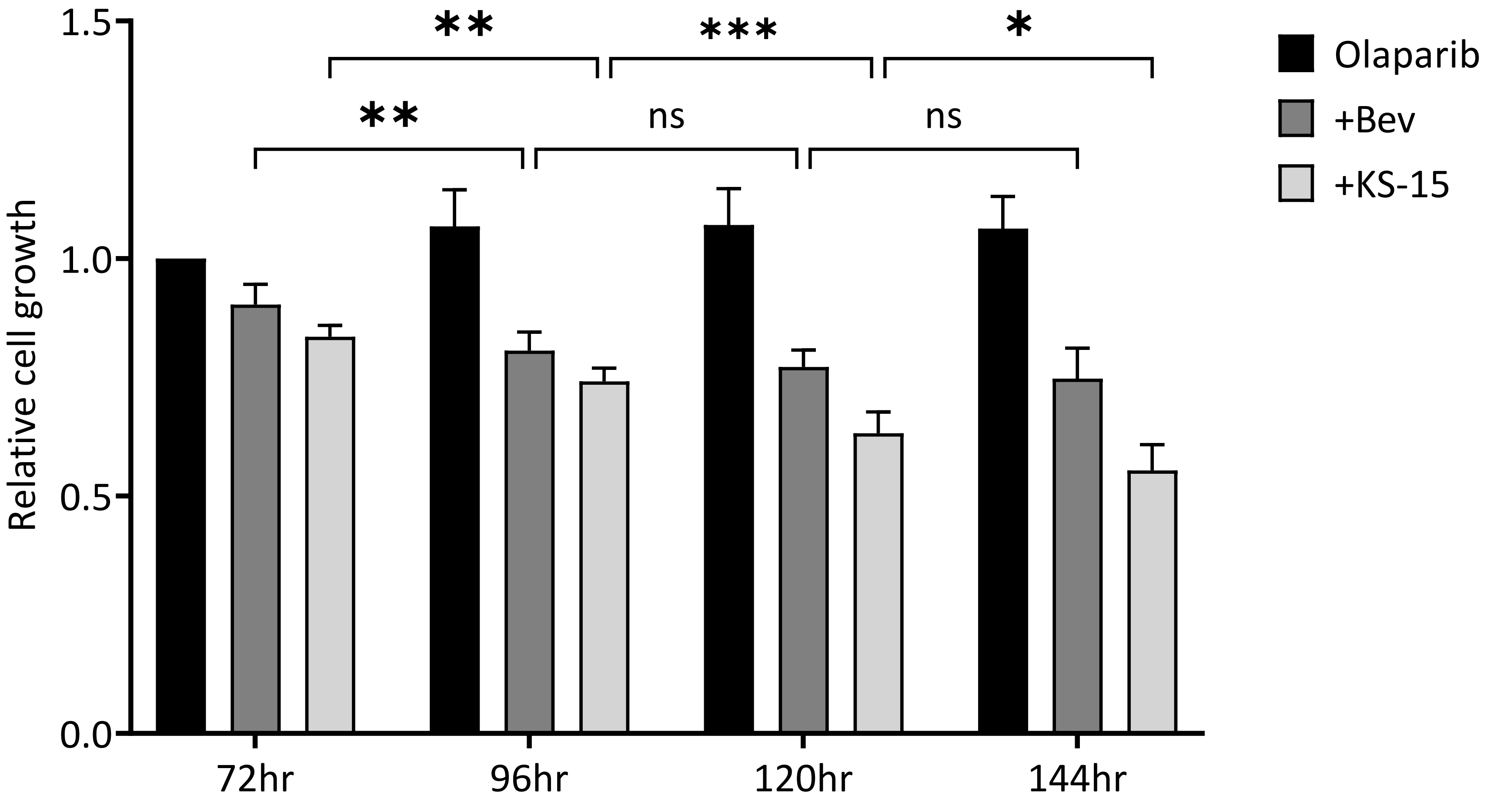

Supplementary Fig. S5. Change in cell growth for each group by time using the MTS assay, using the absorbance at 72 h after olaparib addition as a control. In the olaparib and bevacizumab group, cell growth inhibition reached a plateau at 96 h, whereas in the olaparib and KS-15, an inhibitor of *CRY1*, group, cell growth inhibition was positively correlated with time. Data are shown as mean ± SD. ***$P < 0.001$; **$P < 0.01$; *$P < 0.05$.

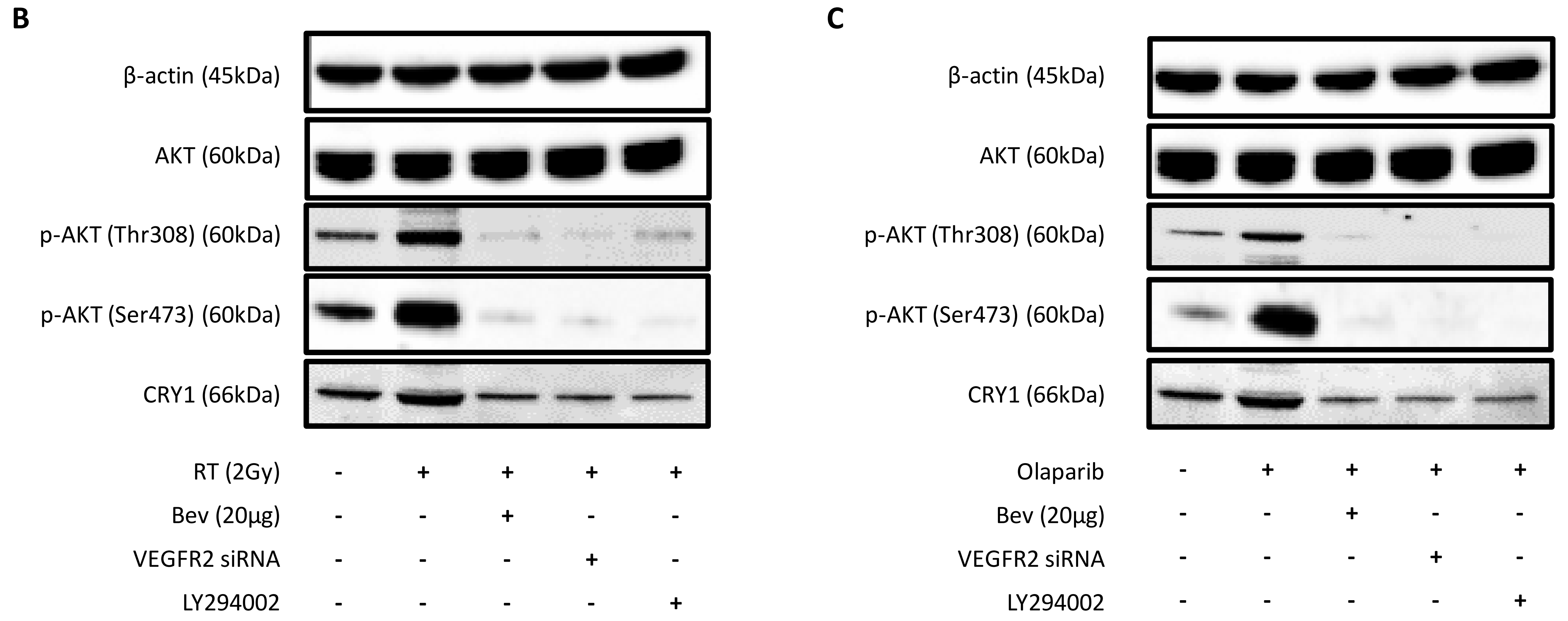

Fig. 3

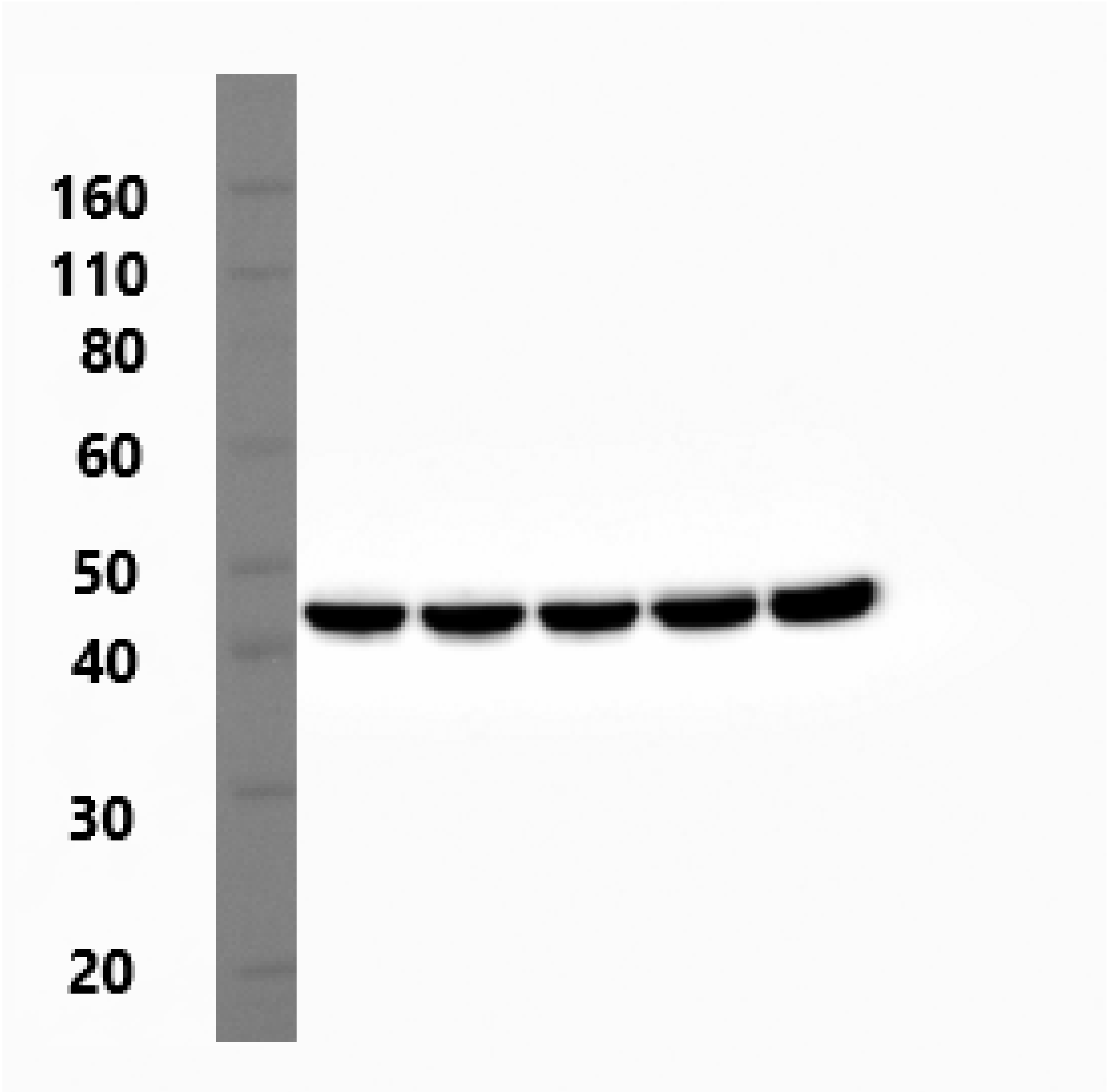

Supplementary Fig. S6. This shows the blotting of β-actin in Figure 3B.

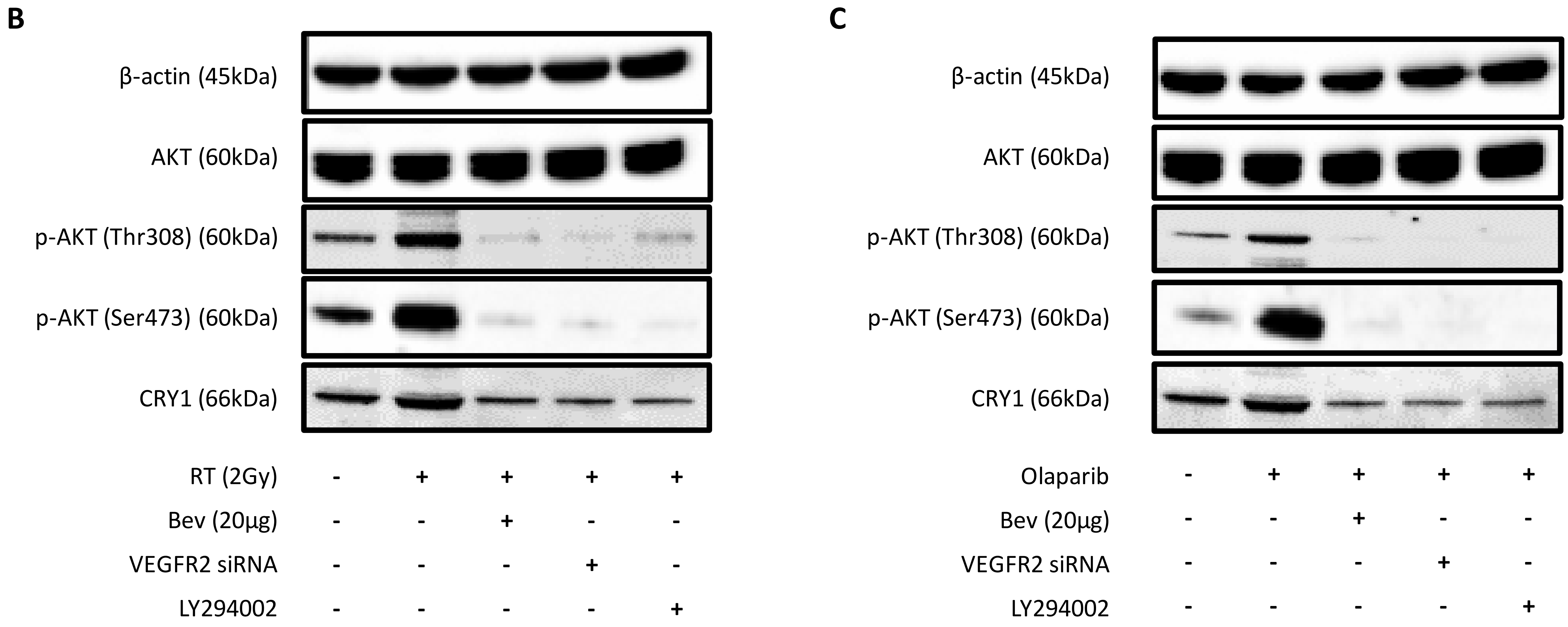

Fig. 3

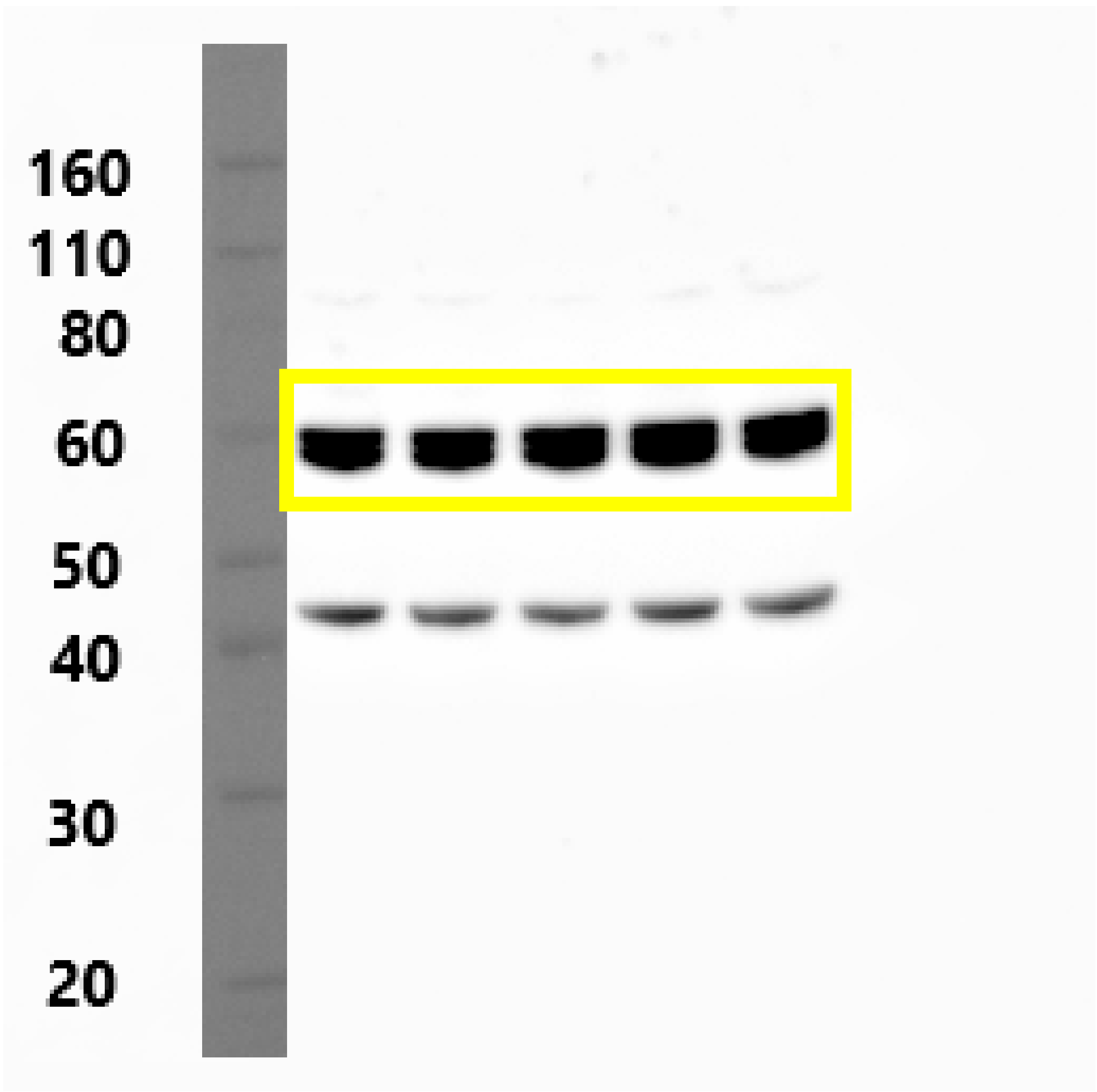

Supplementary Fig. S7. This shows the blotting of AKT in Figure 3B.

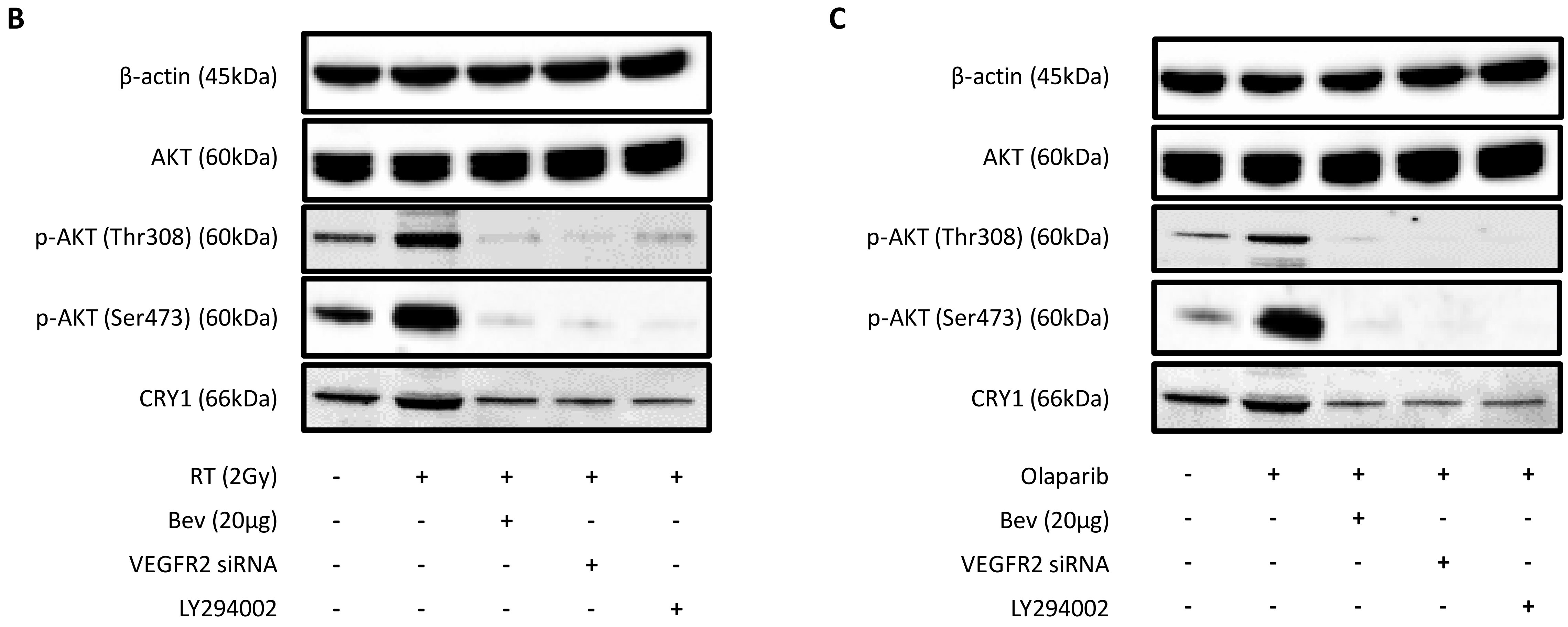

Fig. 3

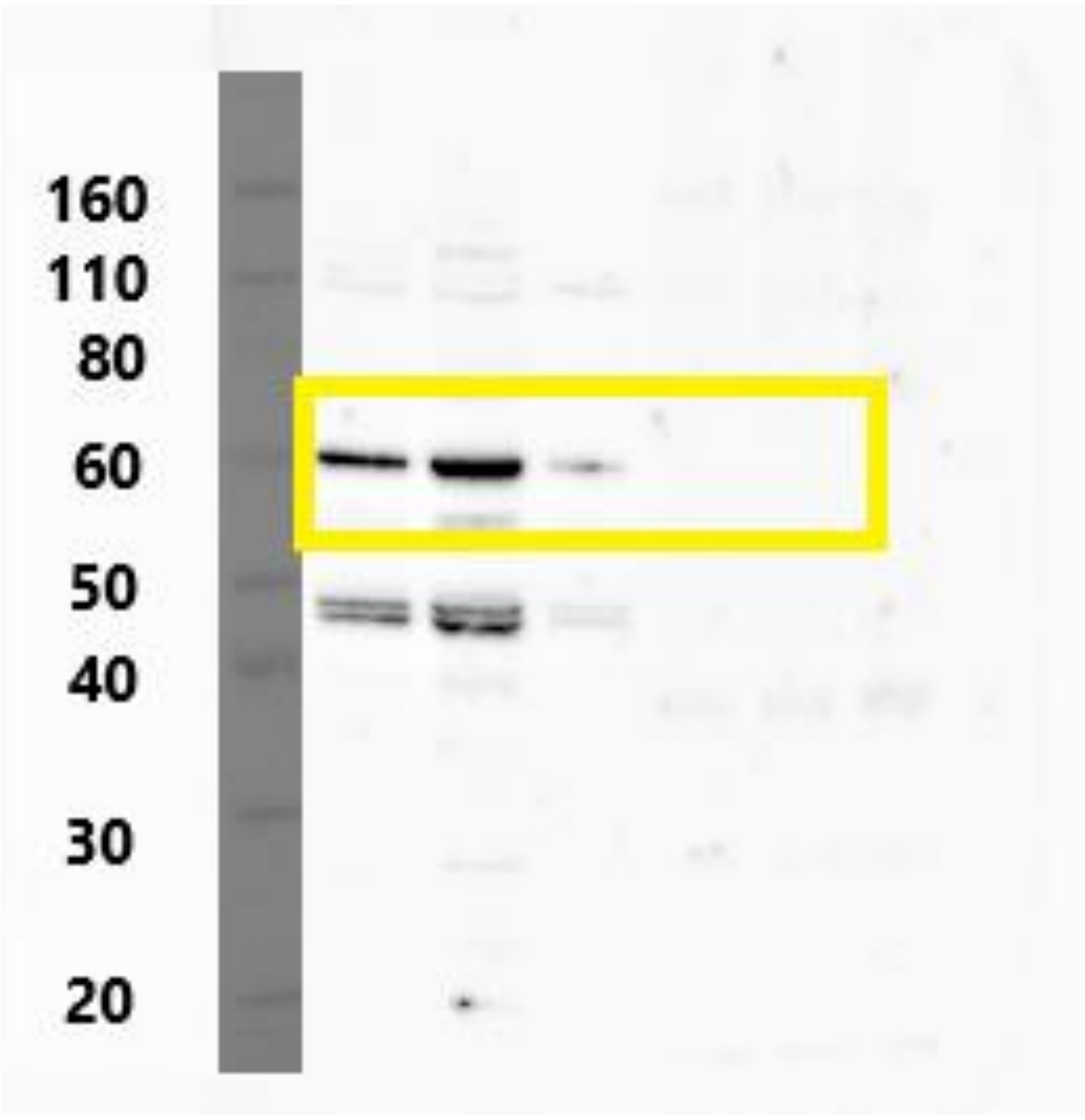

Supplementary Fig. S8. This shows the blotting of p-AKT (Thr308) in Figure 3B.

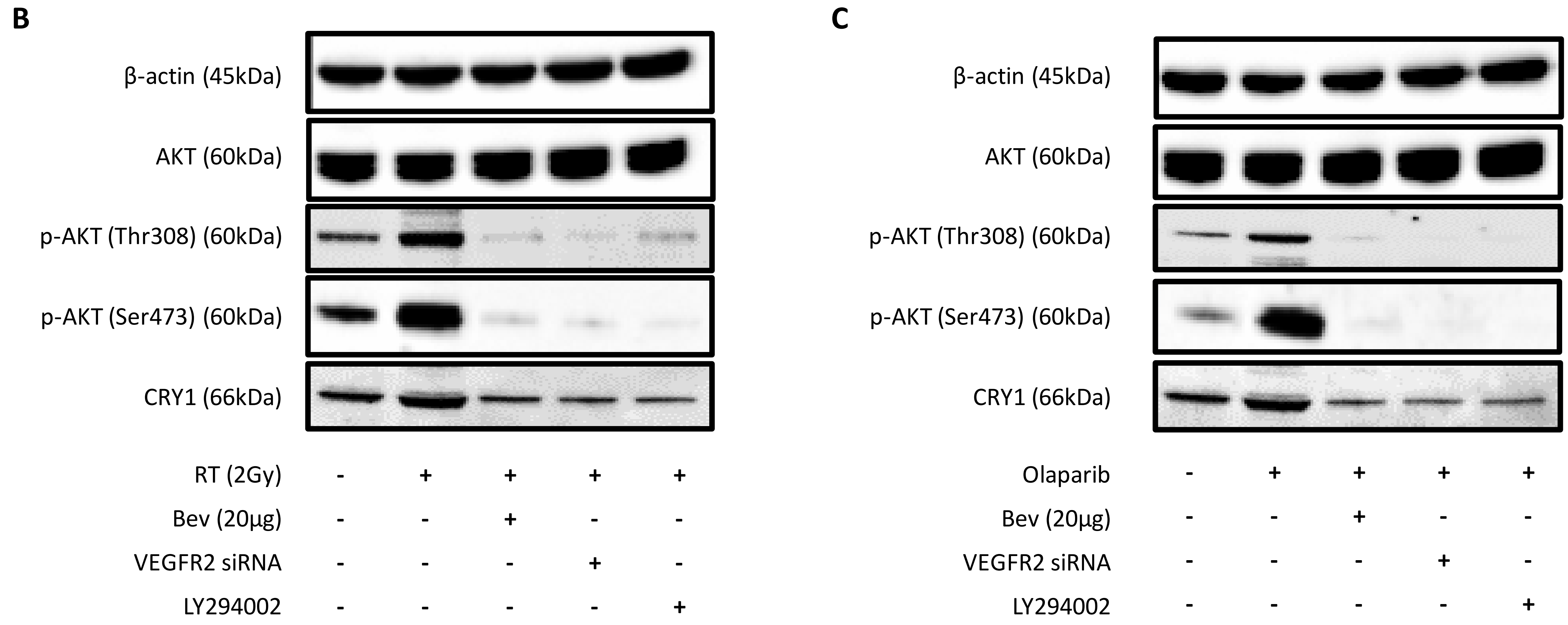

Fig. 3

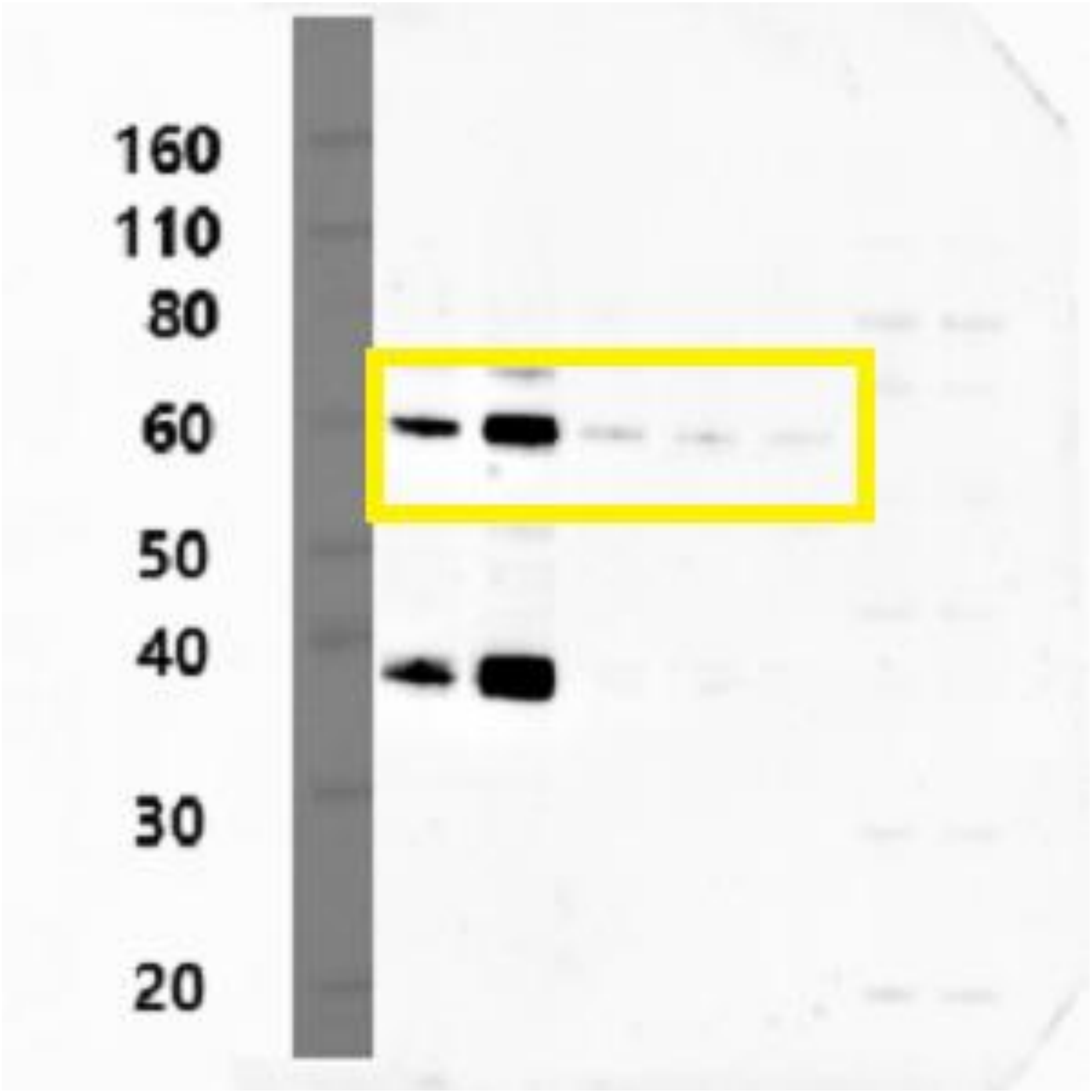

Supplementary Fig. S9. This shows the blotting of p-AKT (Ser473) in Figure 3B.

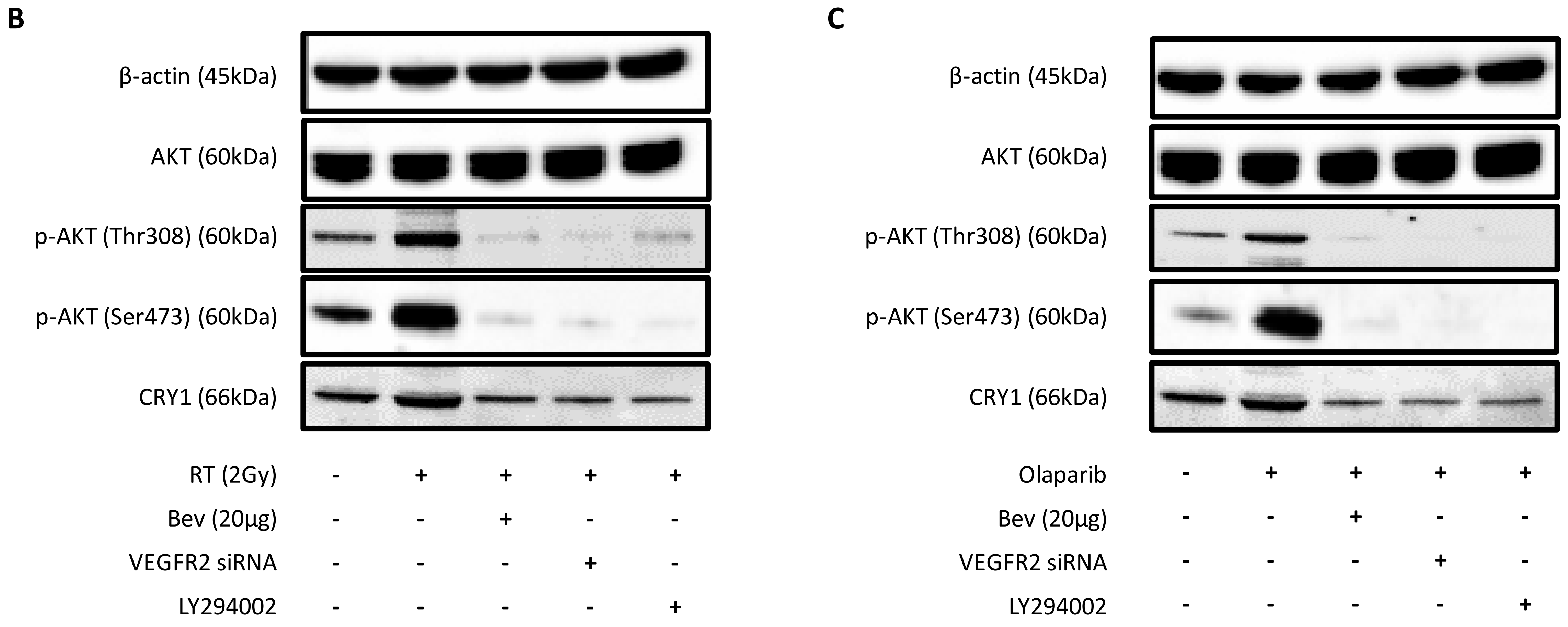

Fig. 3

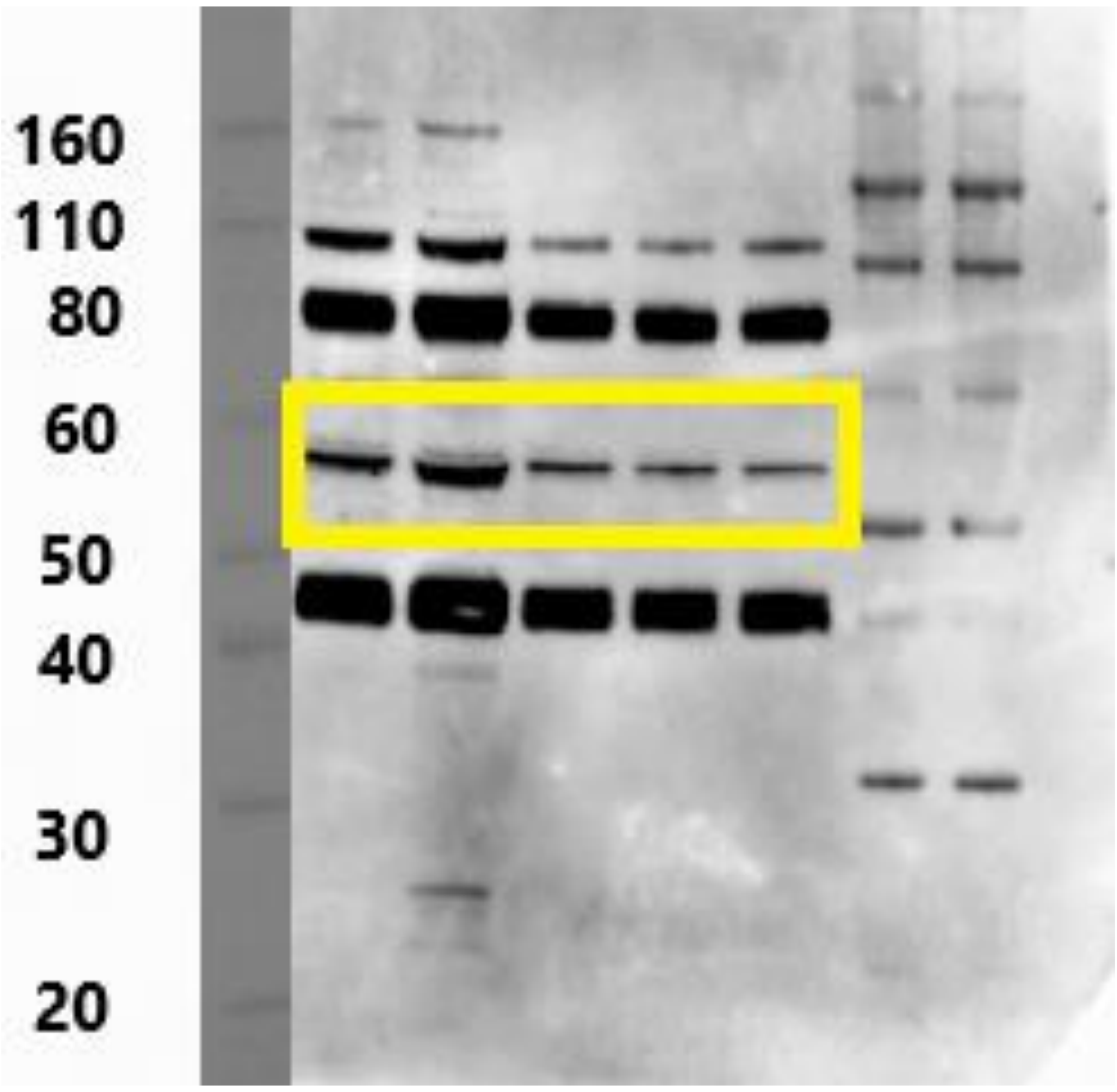

Supplementary Fig. S10. This shows the blotting of CRY1 in Figure 3B.

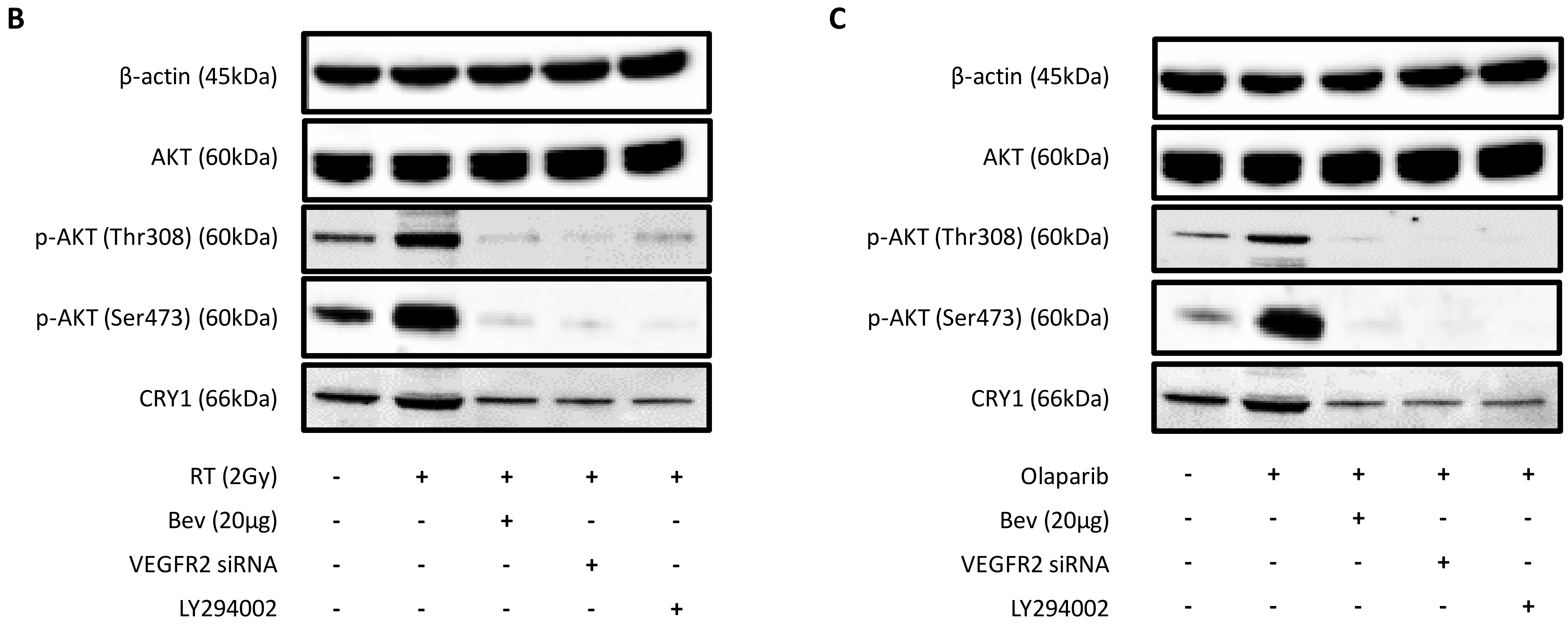

Fig. 3

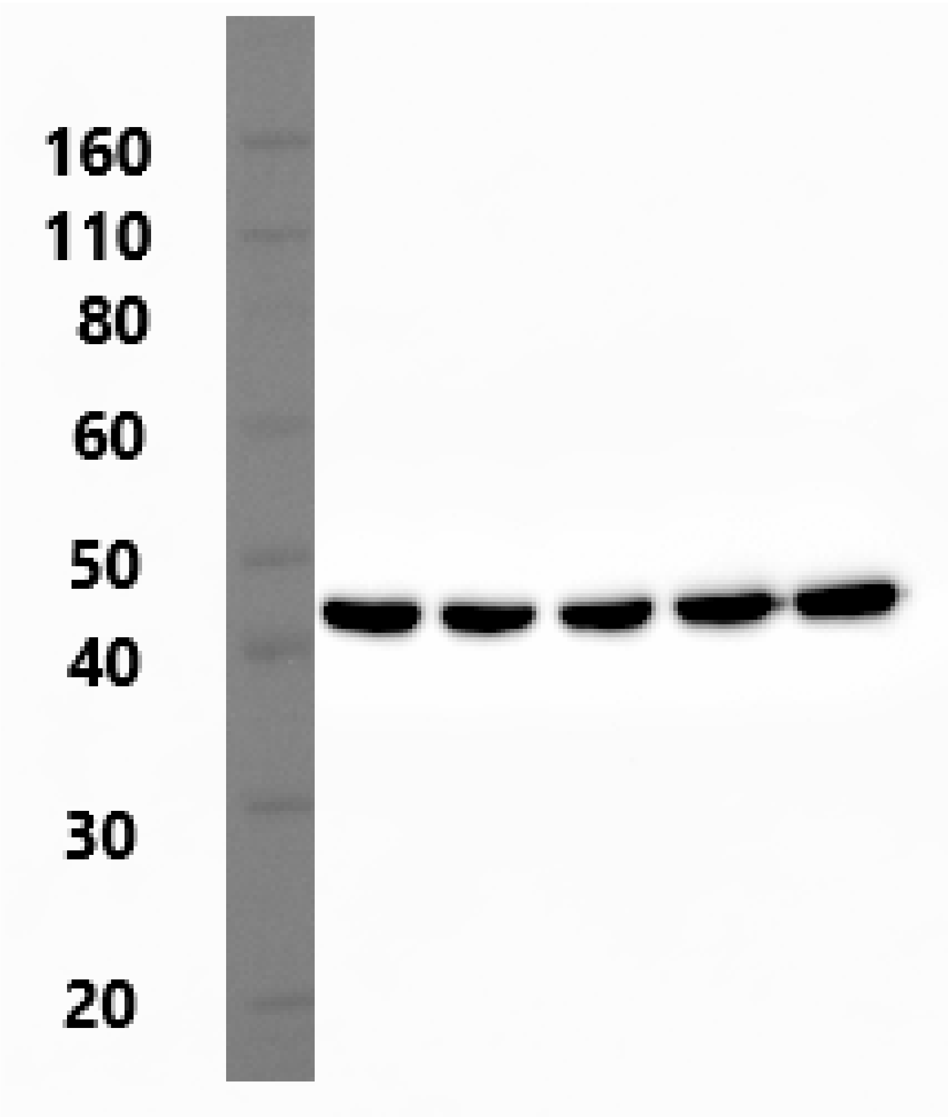

Supplementary Fig. S11. This shows the blotting of β-Actin in Figure 3C.

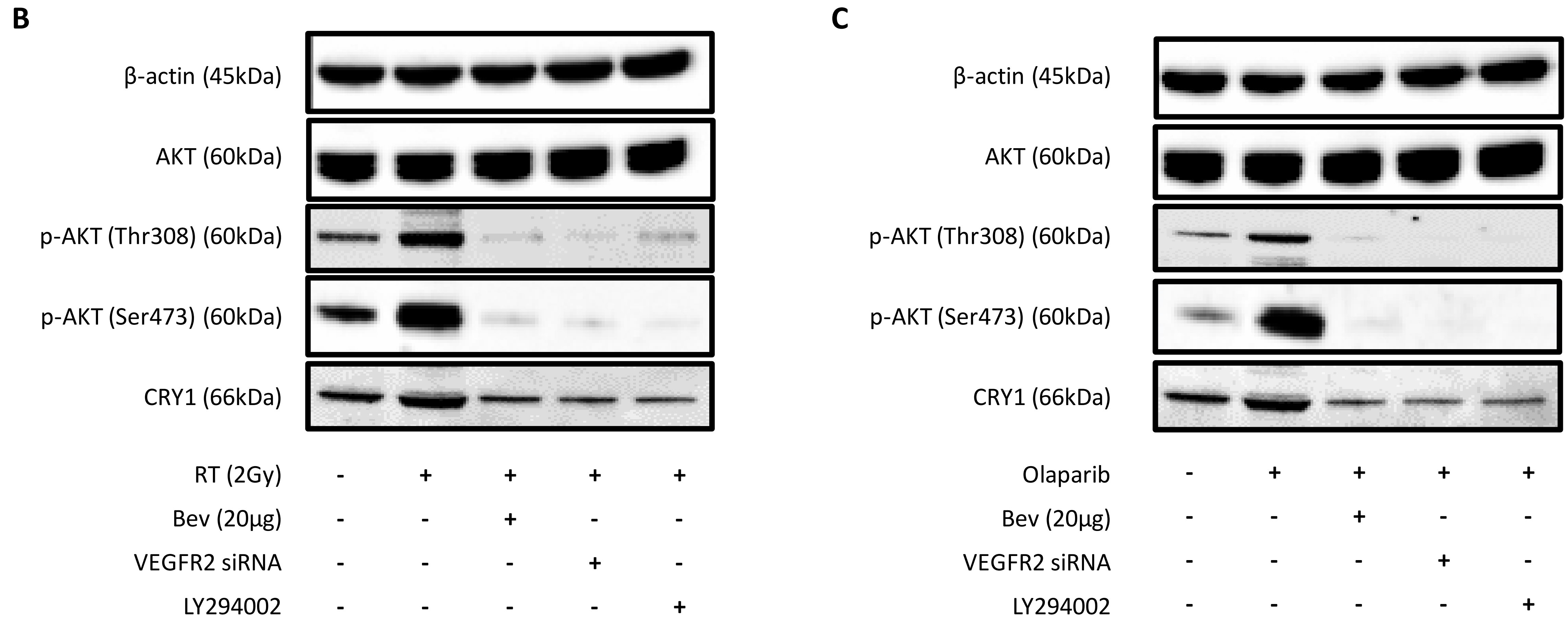

Fig. 3

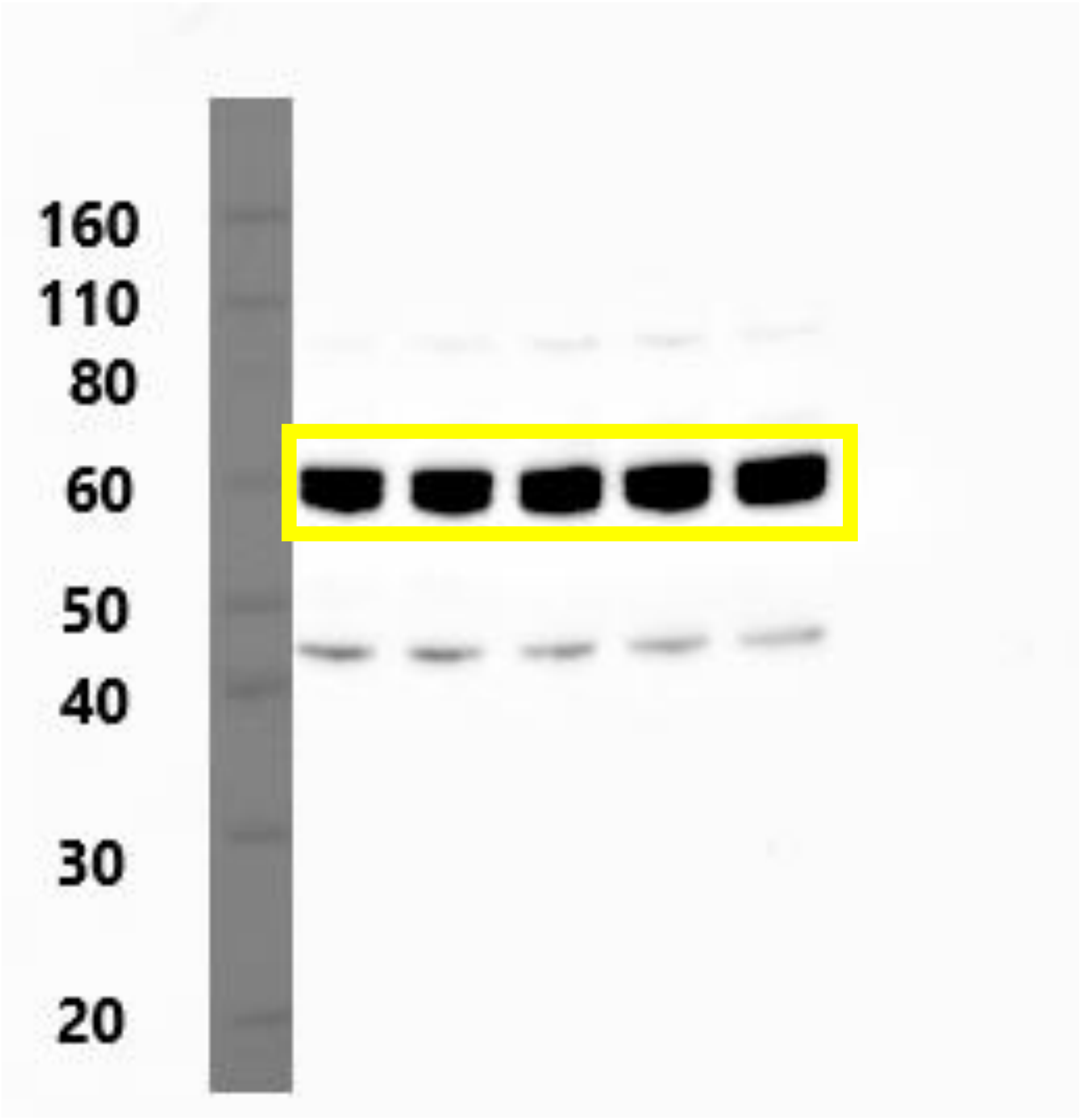

Supplementary Fig. S12. This shows the blotting of AKT in Figure 3C.

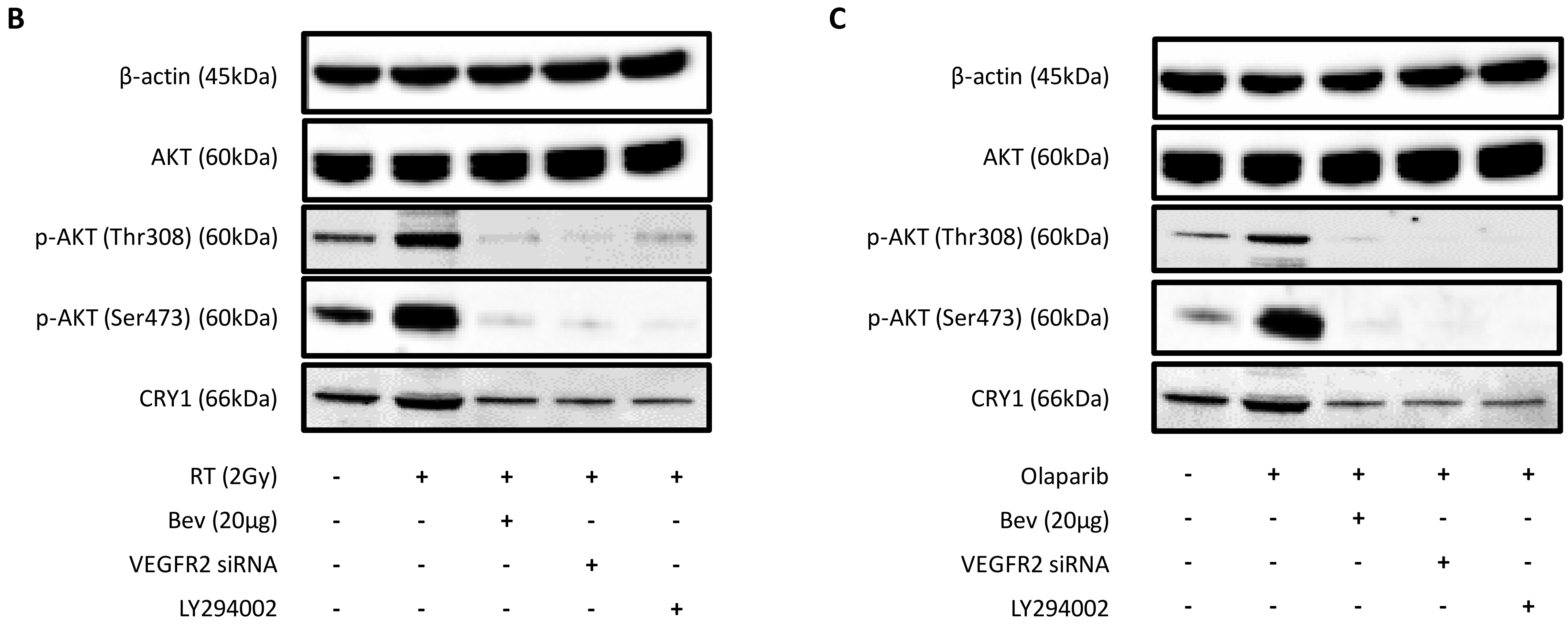

Fig. 3

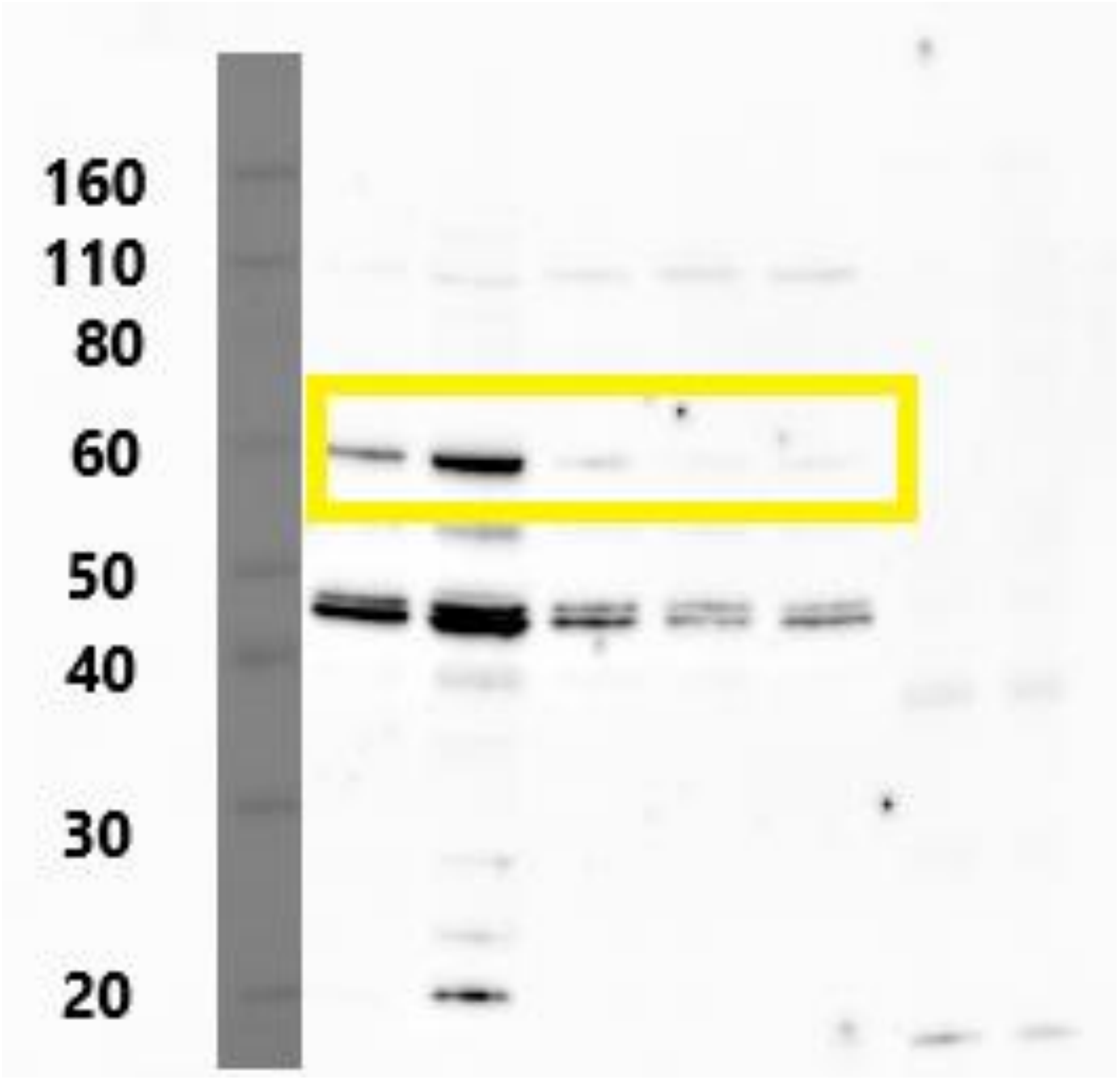

Supplementary Fig. S13. This shows the blotting of p-AKT (Thr308) in Figure 3C.

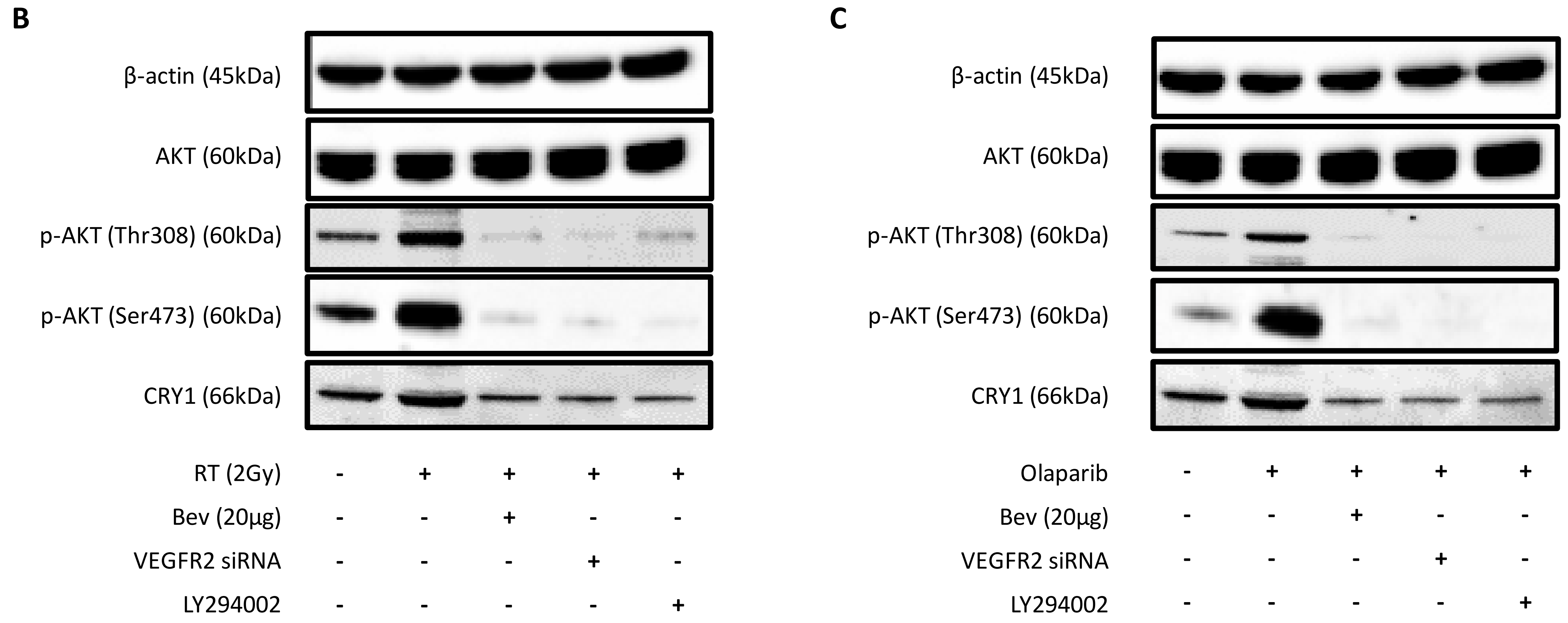

Fig. 3

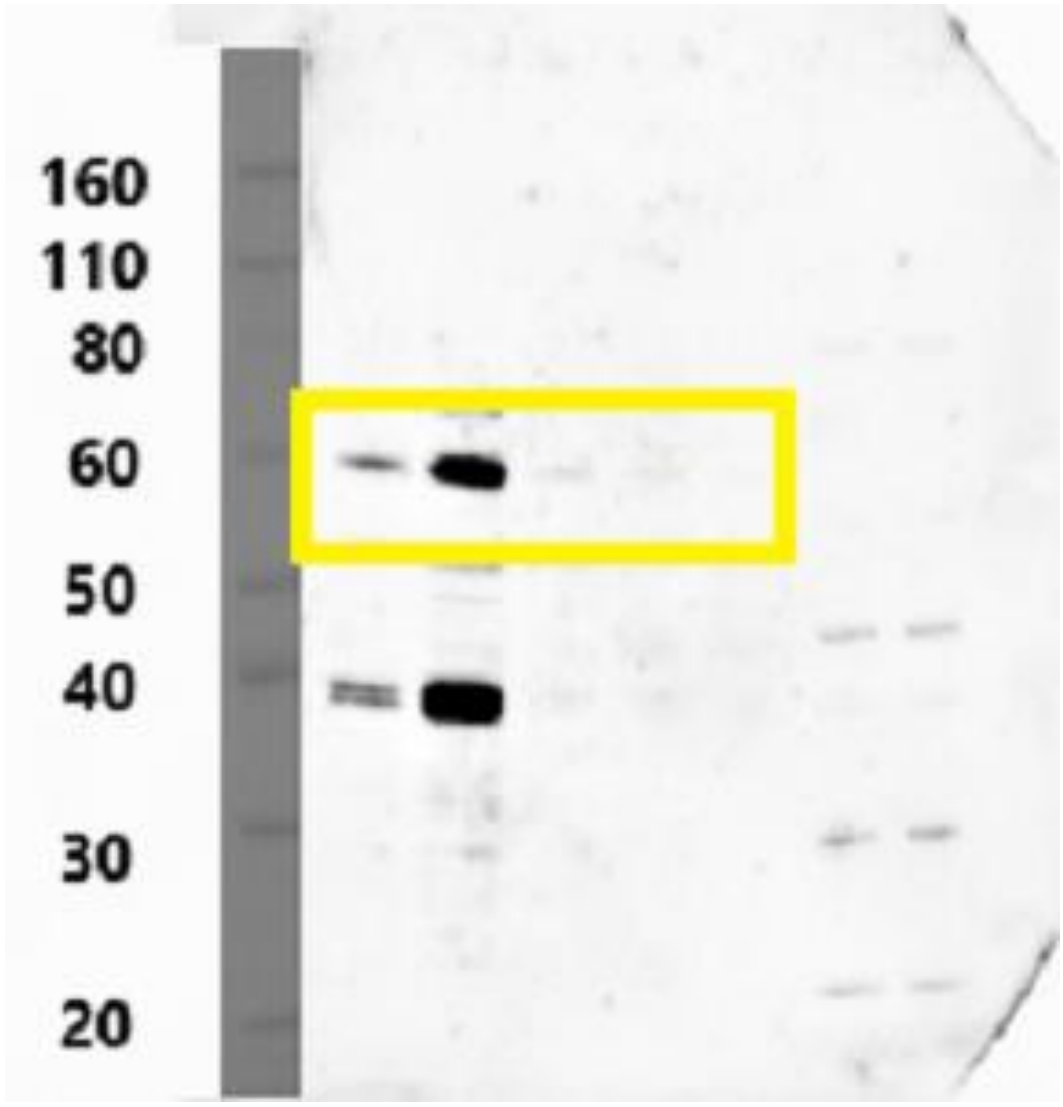

Supplementary Fig. S14. This shows the blotting of p-AKT (Ser473) in Figure 3C.

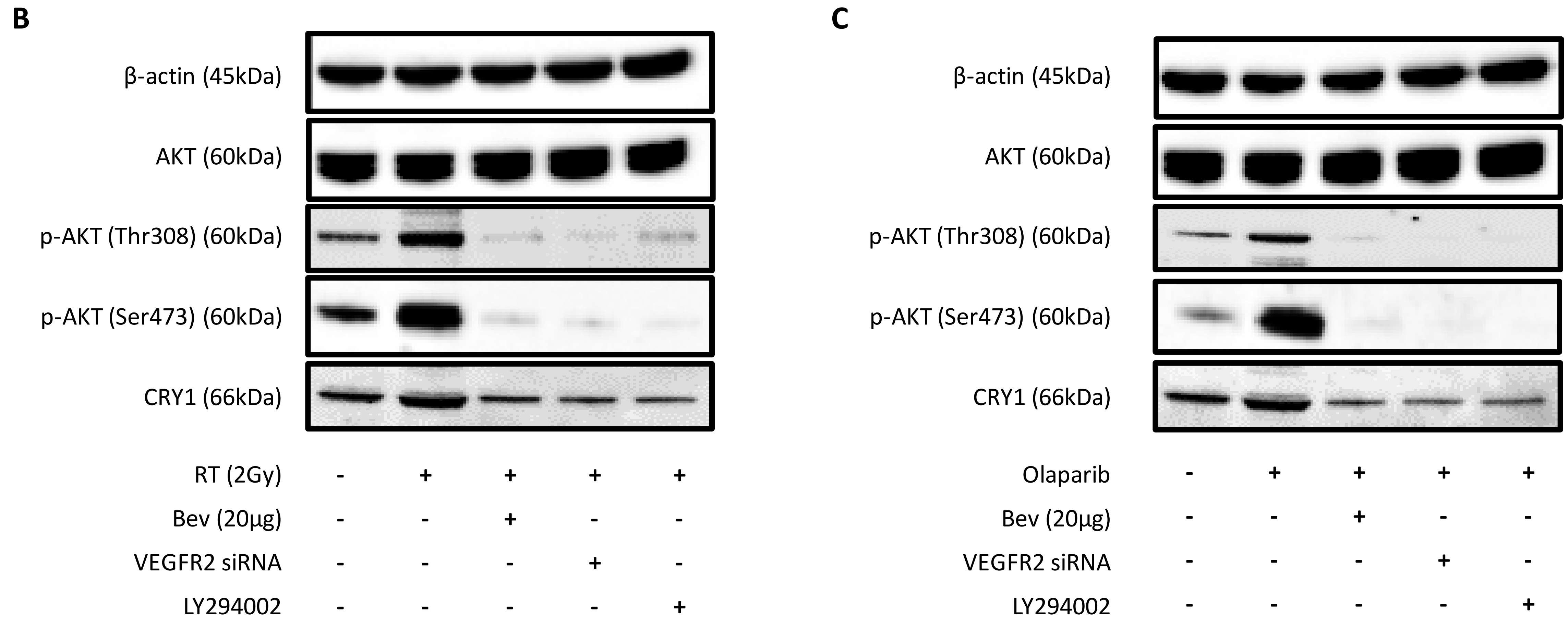

Fig. 3

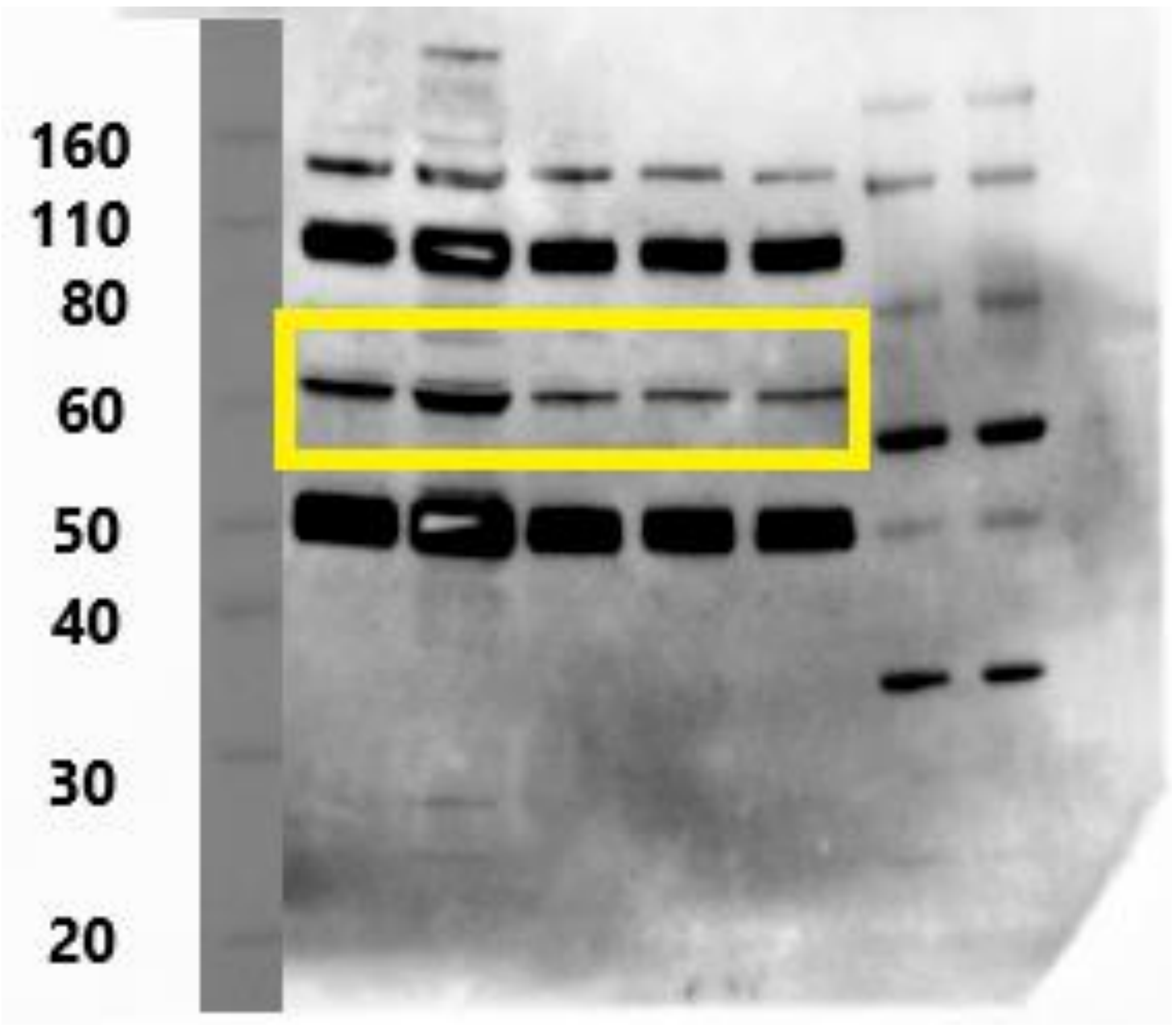

Supplementary Fig. S15. This shows the blotting of CRY1 in Figure 3C.

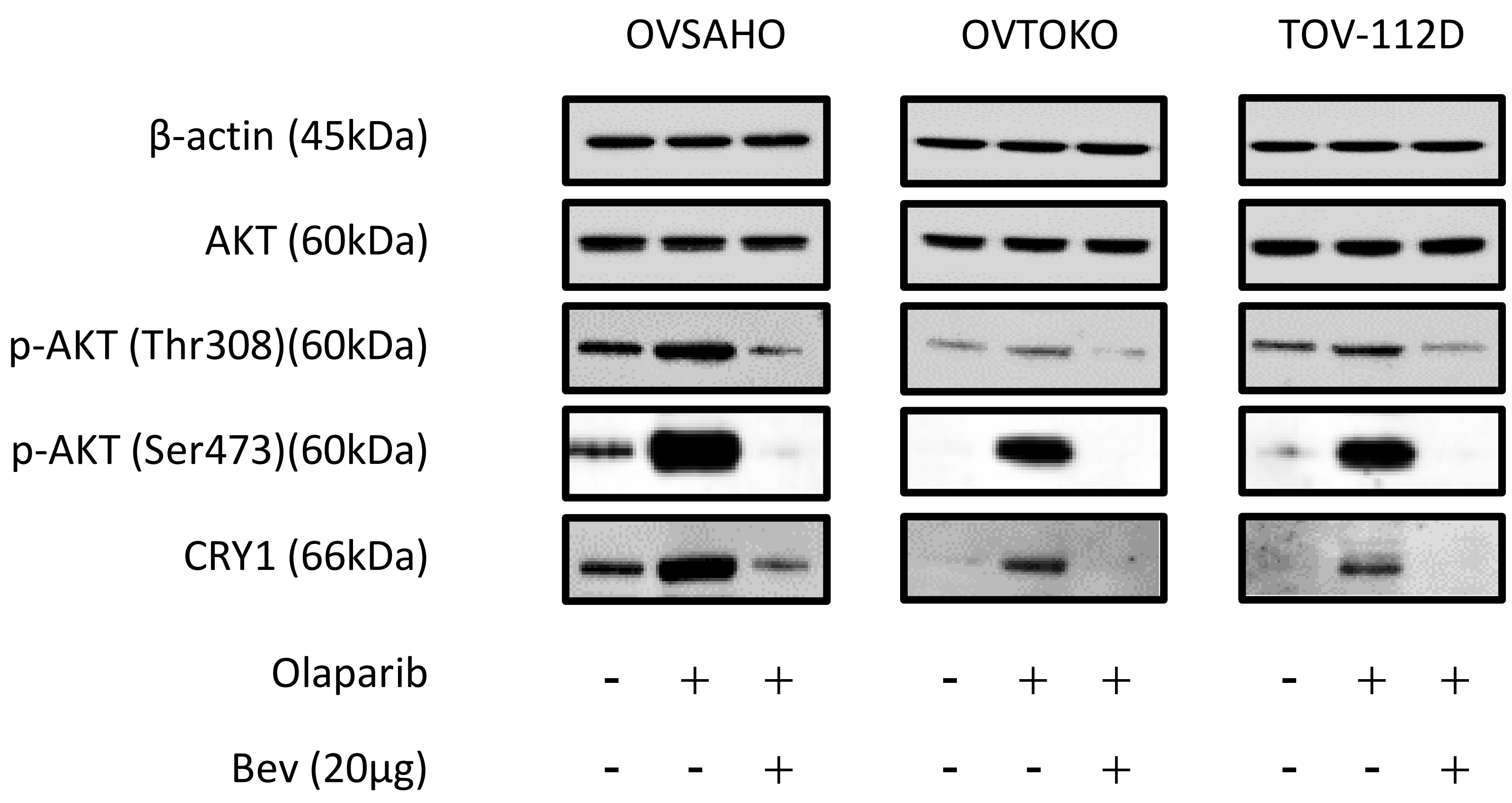

Supplementary Fig. S3.

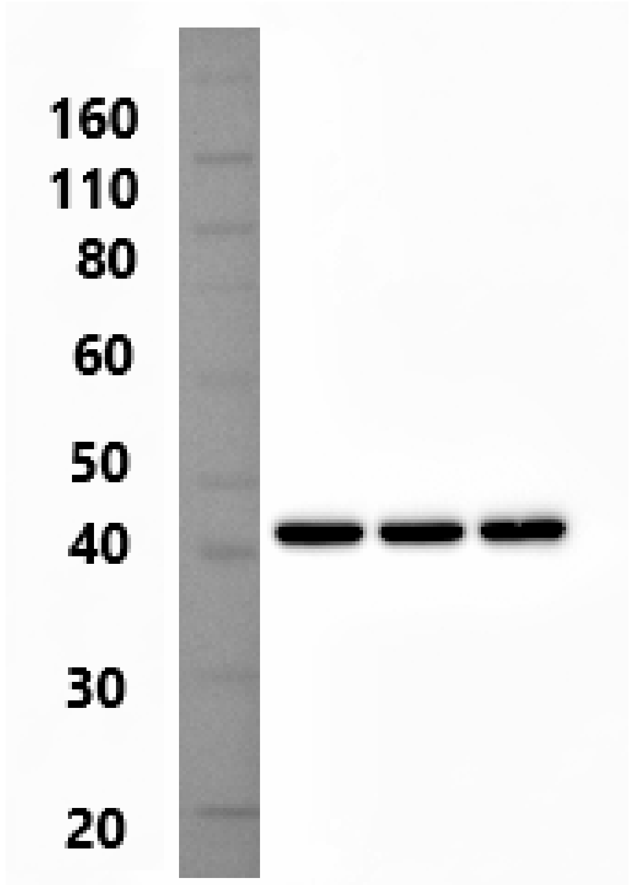

Supplementary Fig. S16. This shows the blotting of β-Actin in OVSAHO in Supplementary Figure S3.

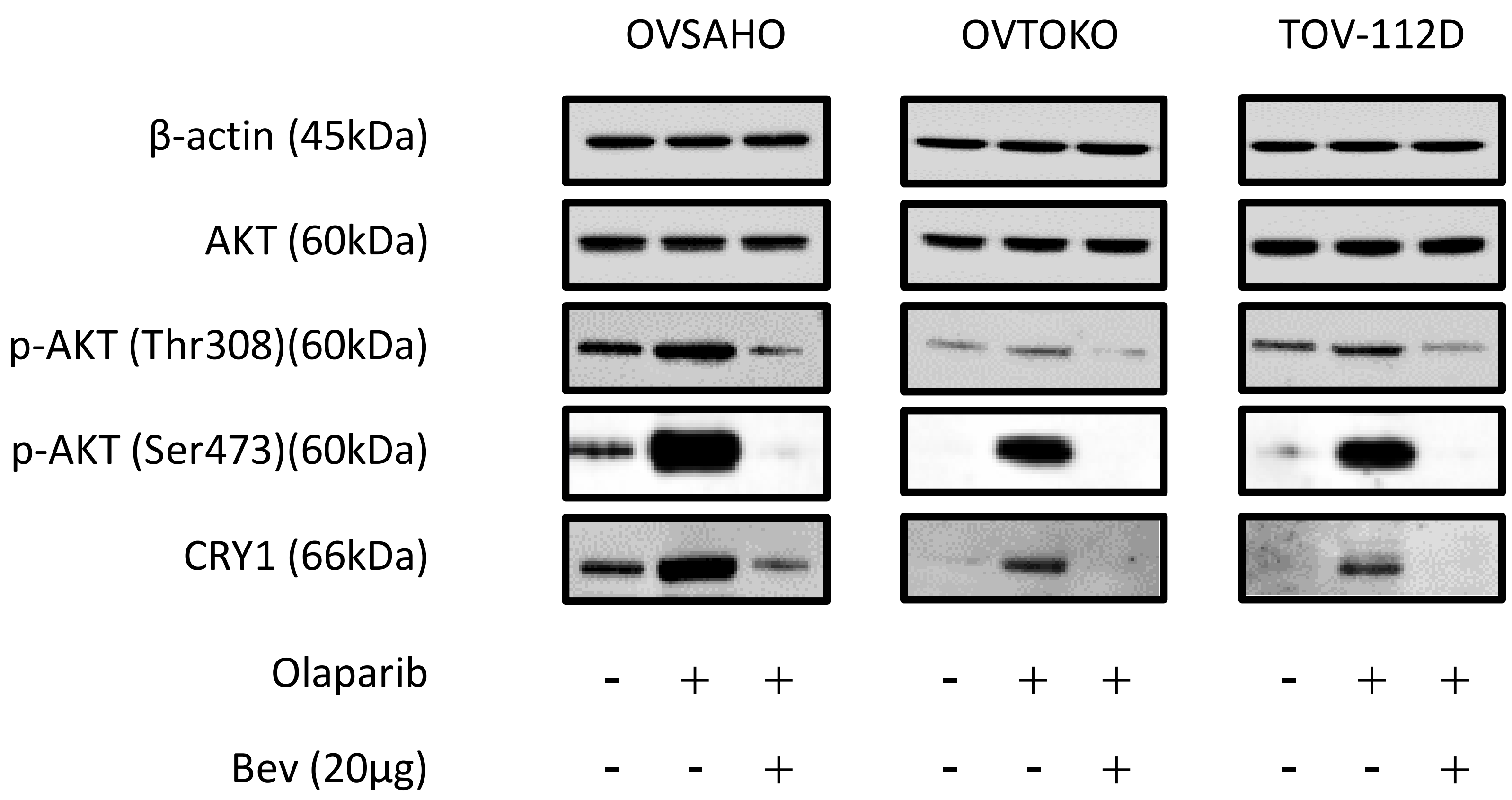

Supplementary Fig. S3.

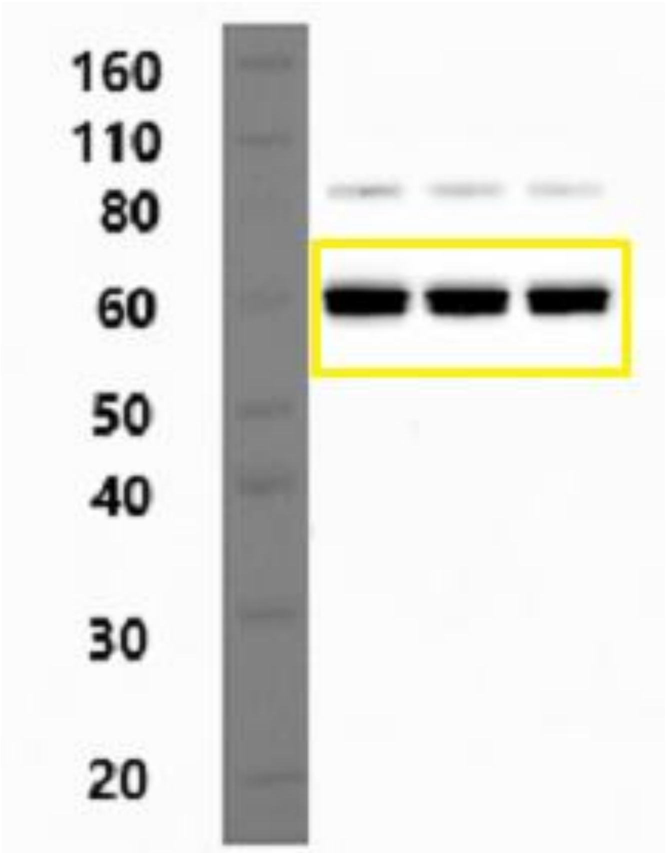

Supplementary Fig. S17. This shows the blotting of AKT in OVSAHO in Supplementary Figure S3.

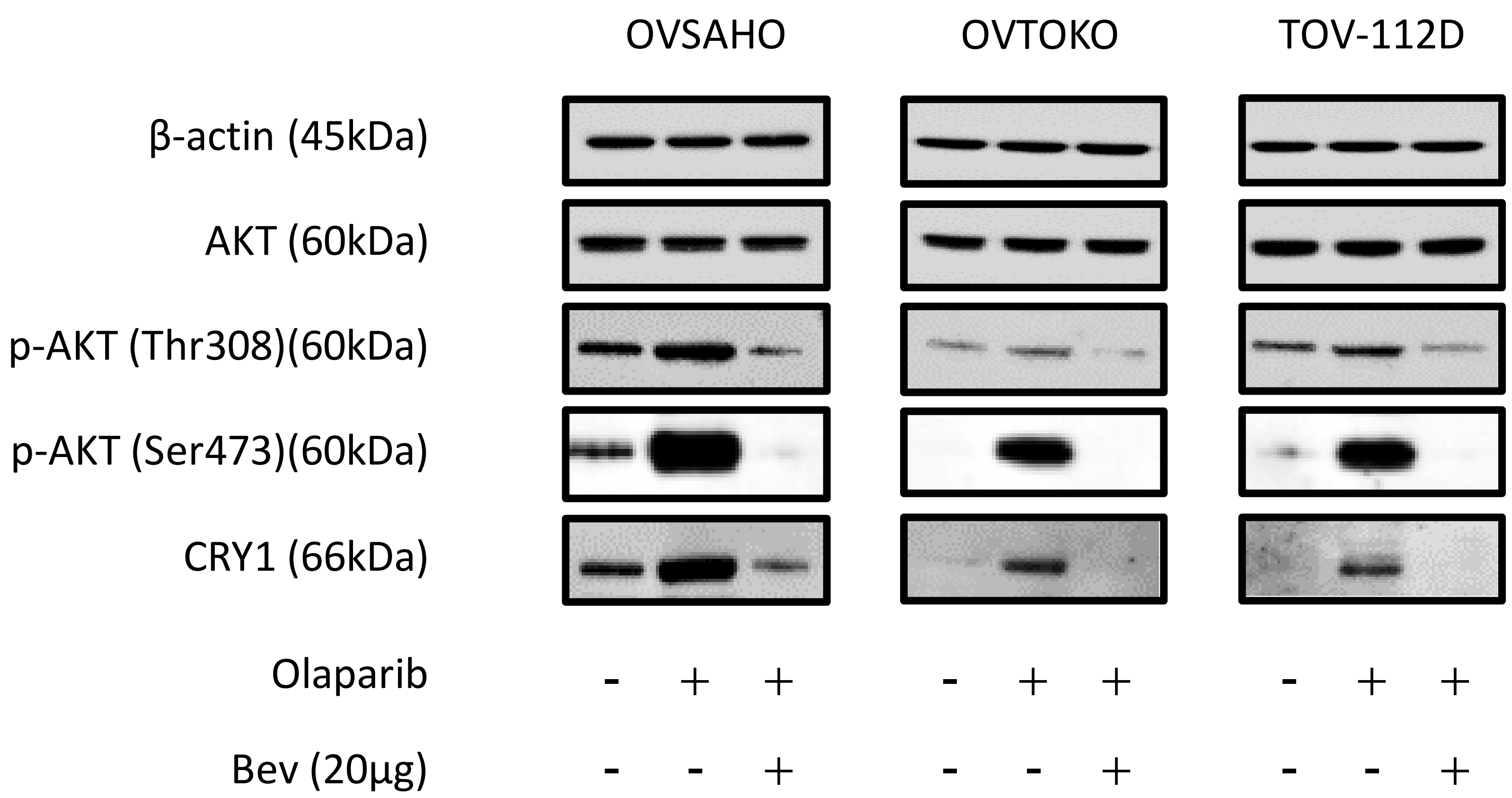

Supplementary Fig. S3.

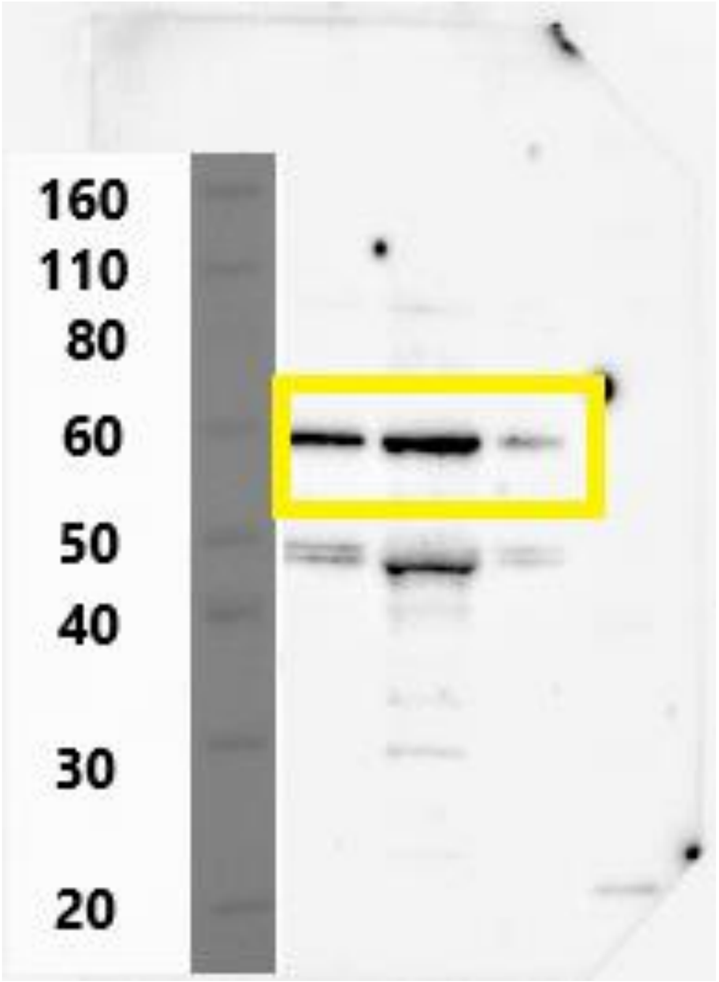

Supplementary Fig. S18. This shows the blotting of p-AKT (Thr308) in OVSAHO in Supplementary Figure S3.

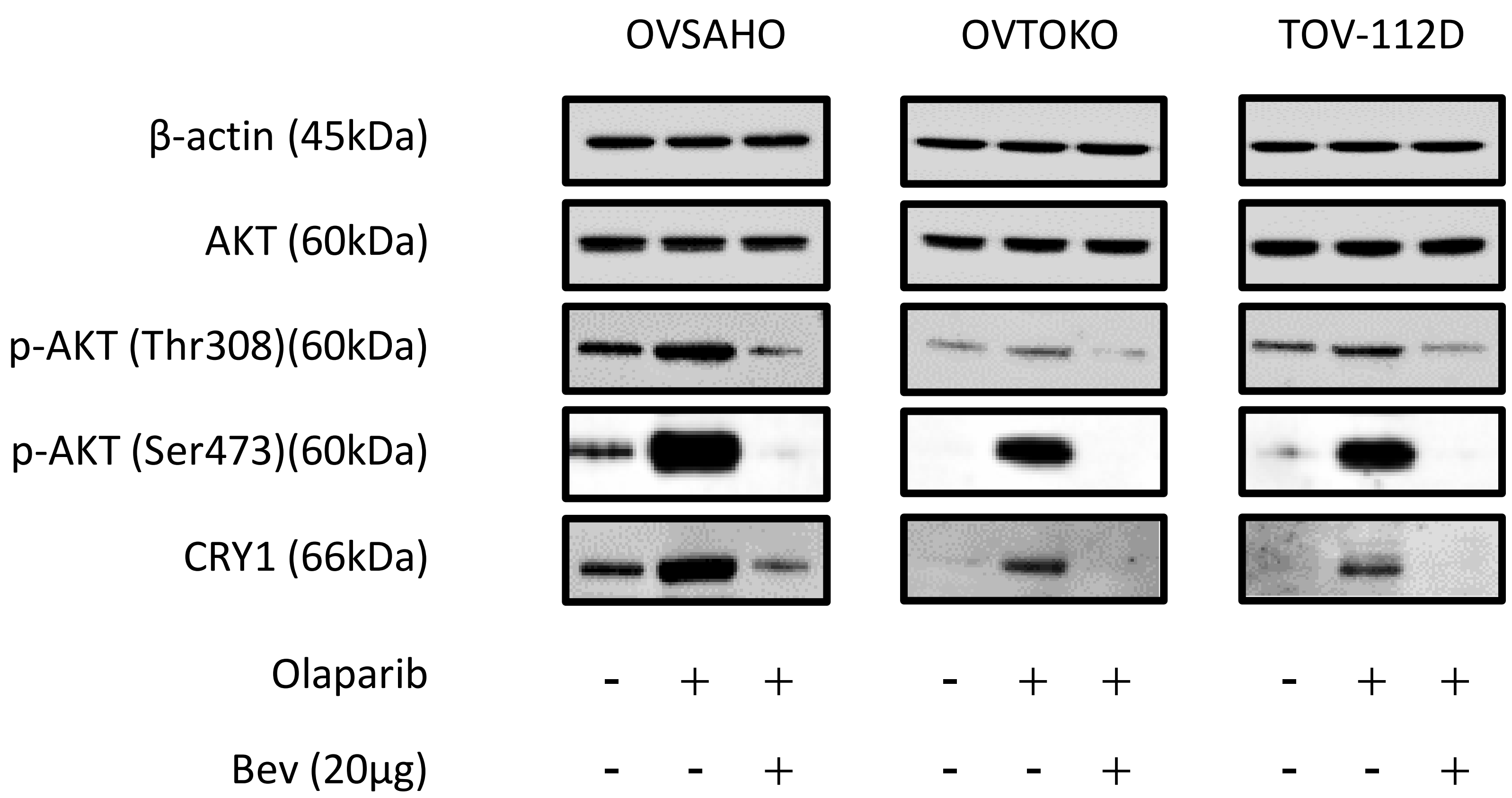

Supplementary Fig. S3.

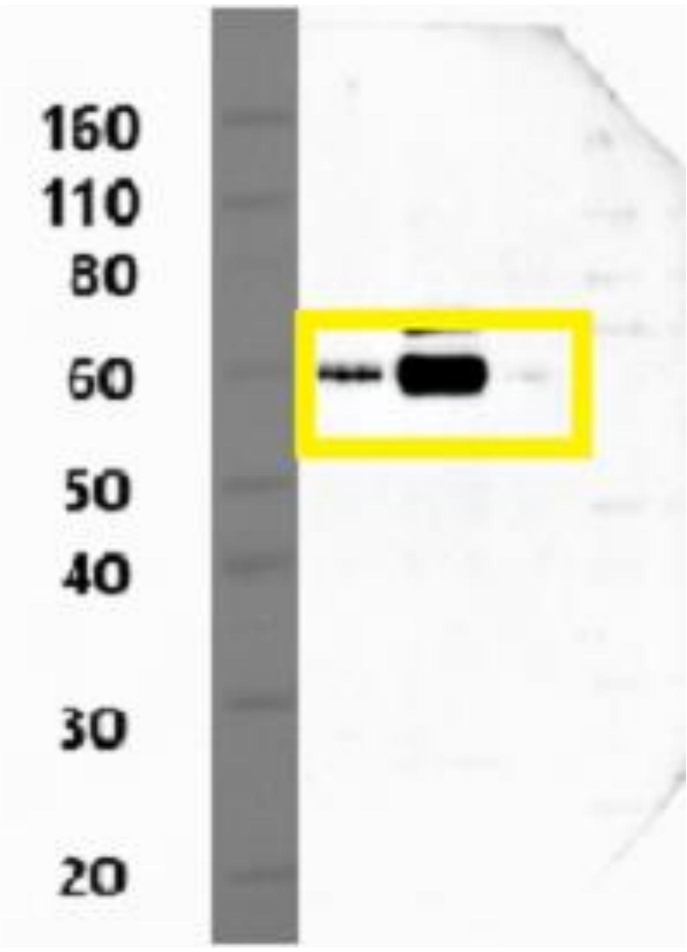

Supplementary Fig. S19. This shows the blotting of p-AKT (Ser473) in OVSAHO in Supplementary Figure S3.

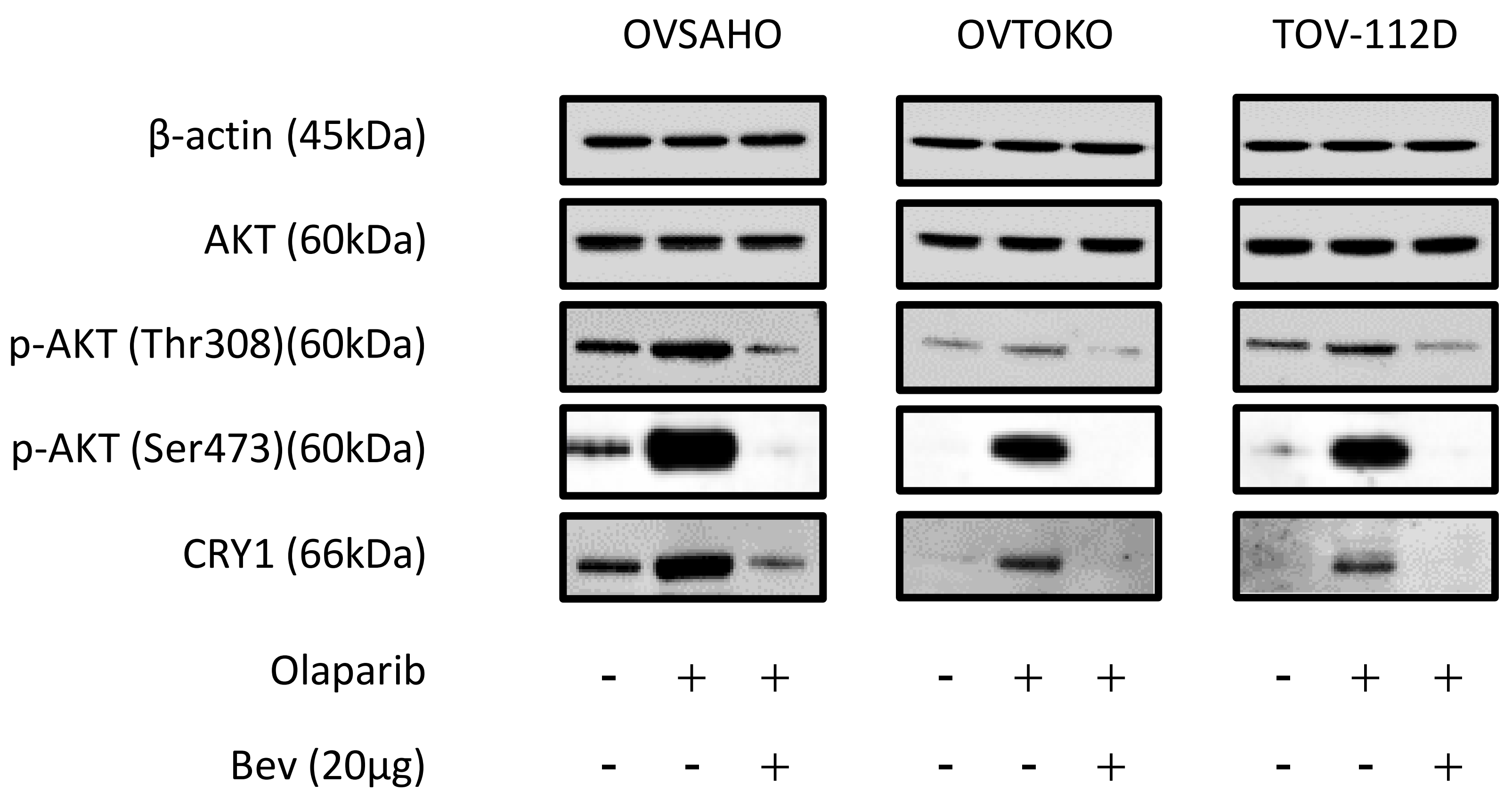

Supplementary Fig. S3.

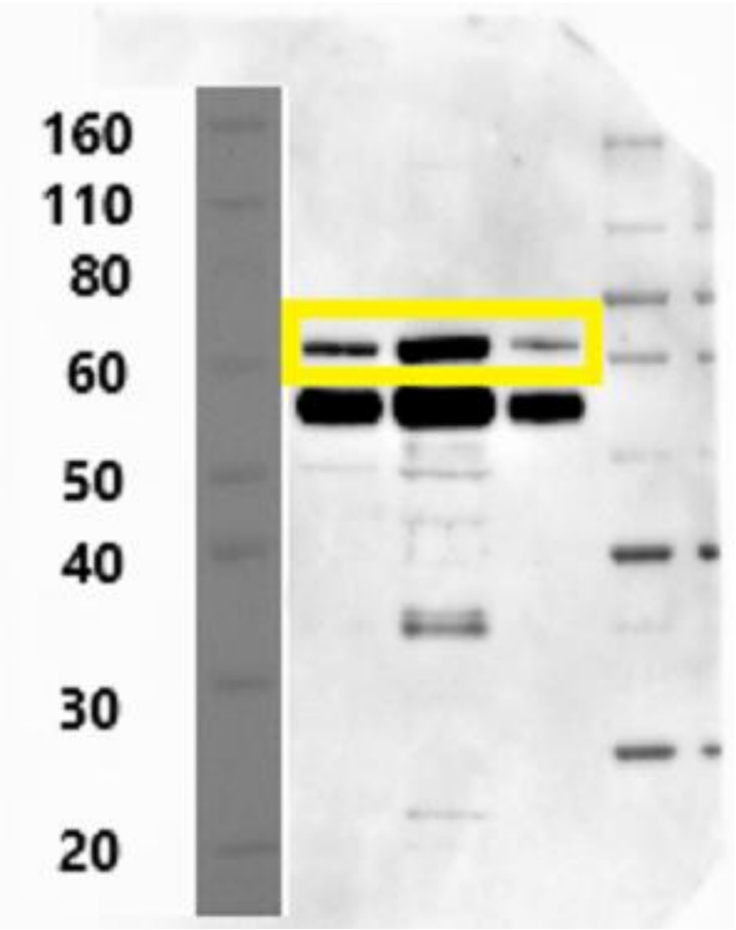

Supplementary Fig. S20. This shows the blotting of CRY1 in OVSAHO in Supplementary Figure S3.

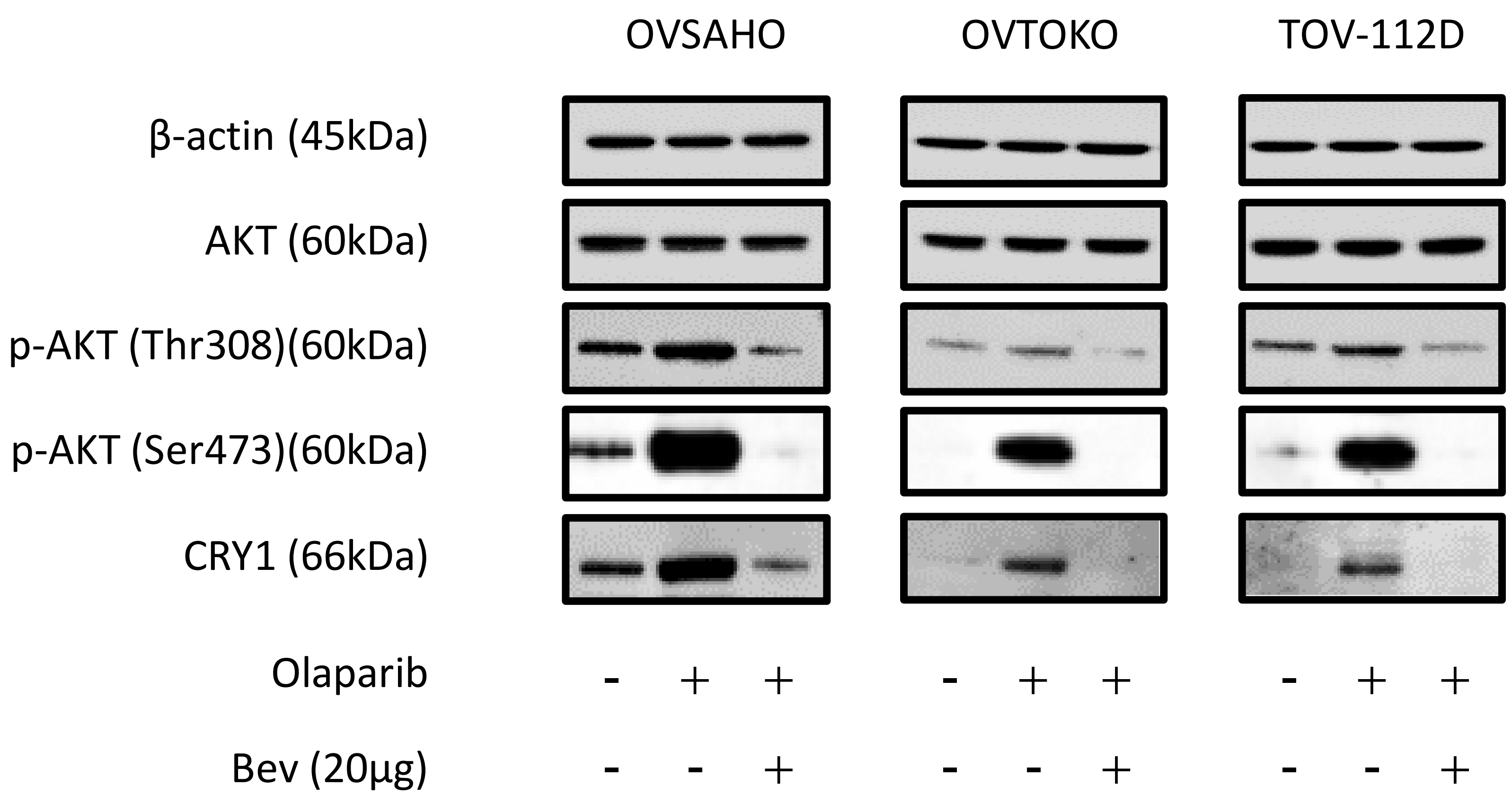

Supplementary Fig. S3.

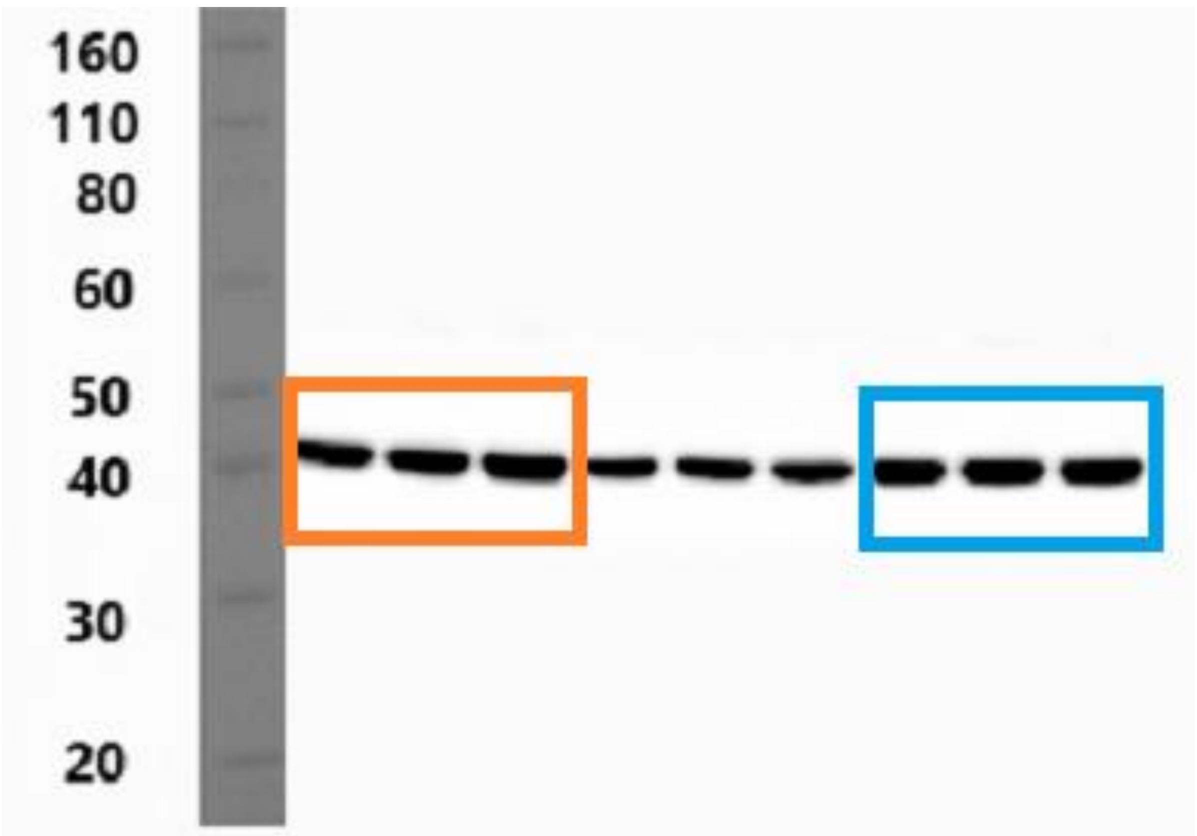

Supplementary Fig. S21. This shows the blotting of β-Actin in OVTOKO (orange) and TOV-112D (blue) in Supplementary Figure S3.

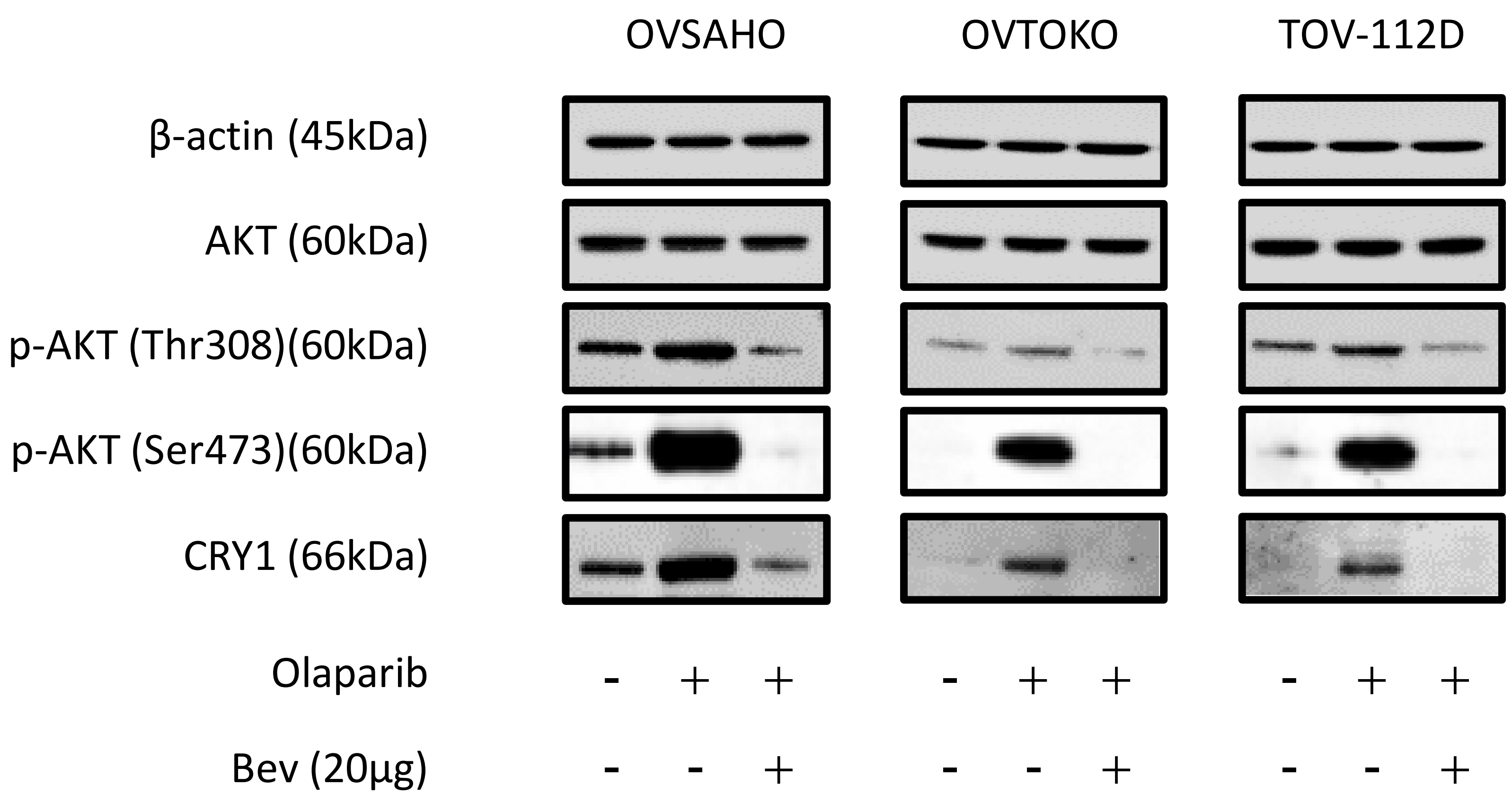

Supplementary Fig. S3.

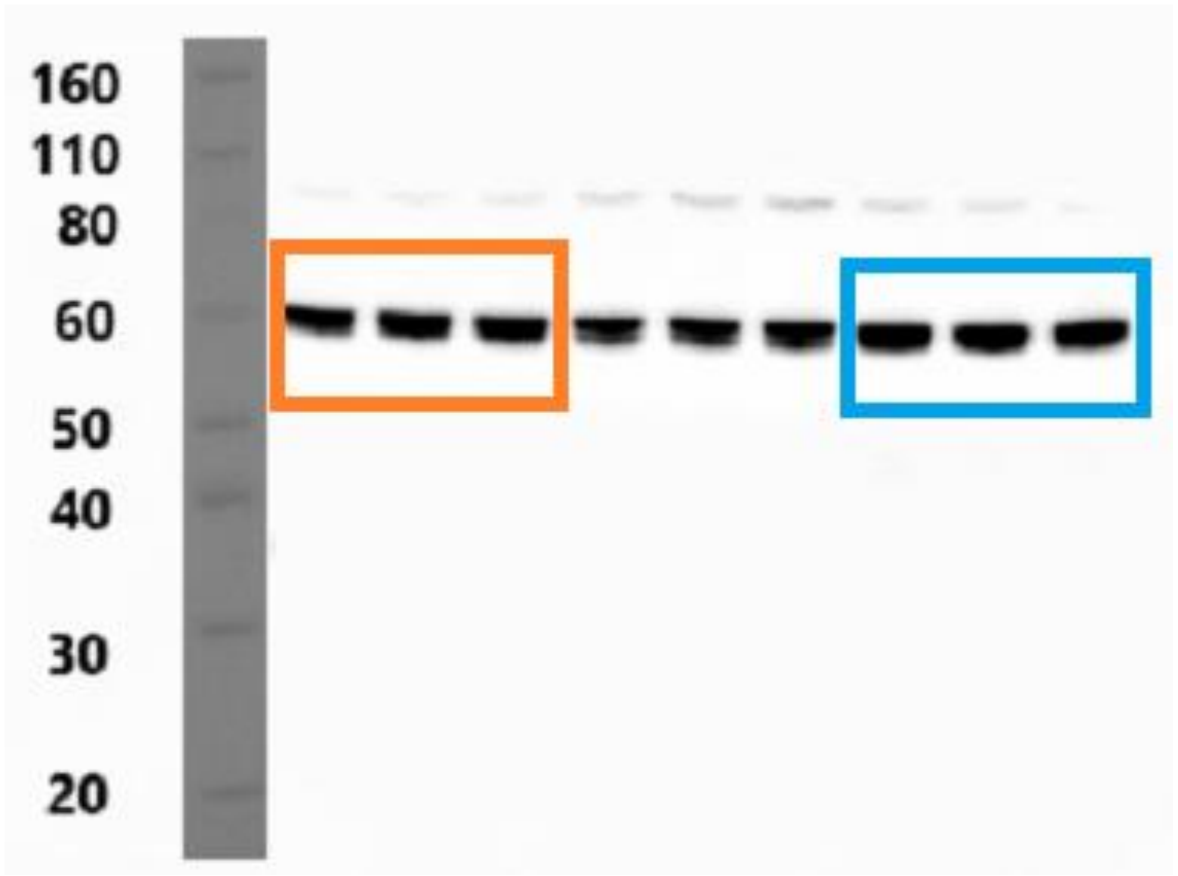

Supplementary Fig. S22. This shows the blotting of AKT in OVTOKO (orange) and TOV-112D (blue) in Supplementary Figure S3.

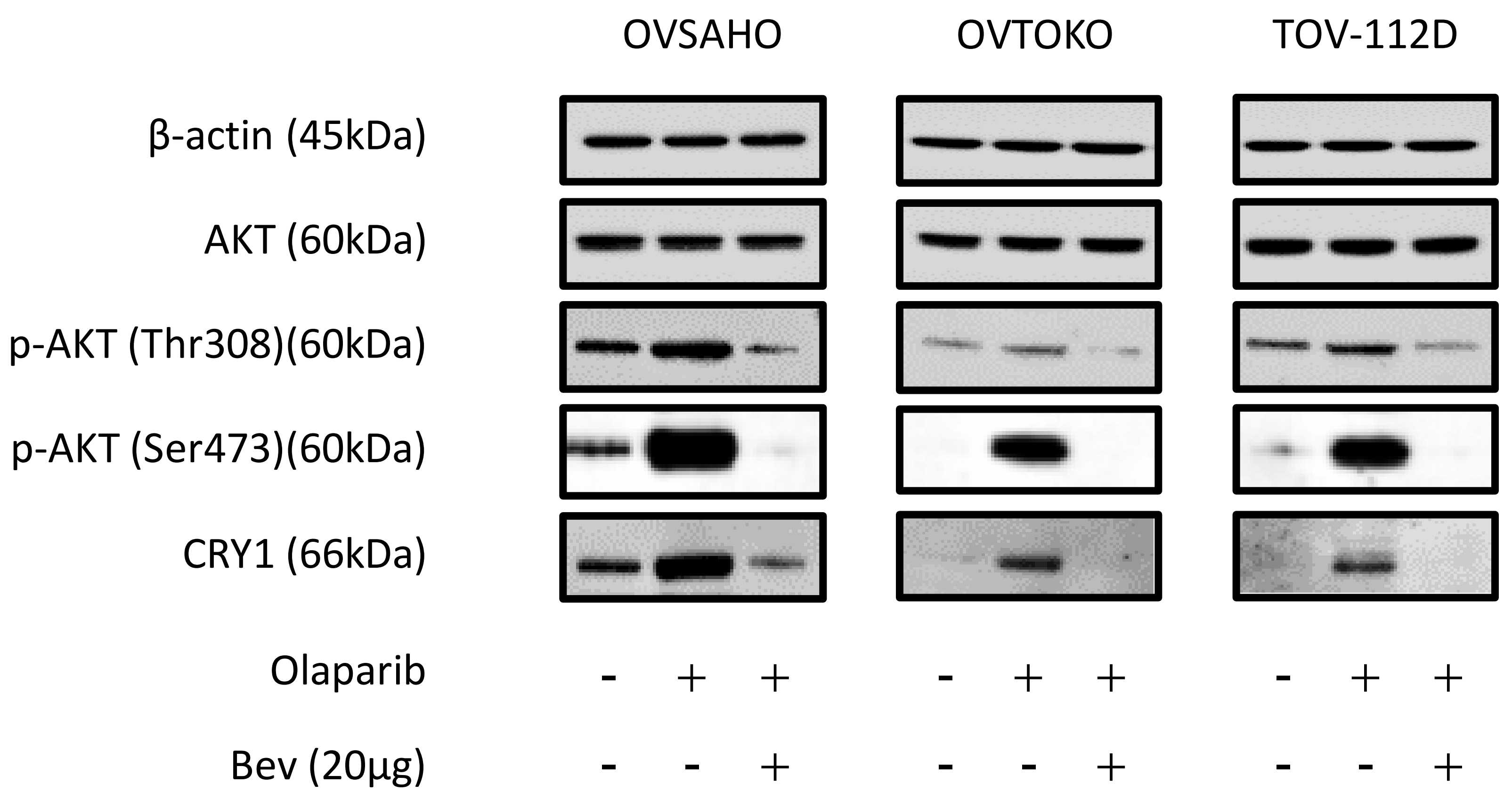

Supplementary Fig. S3.

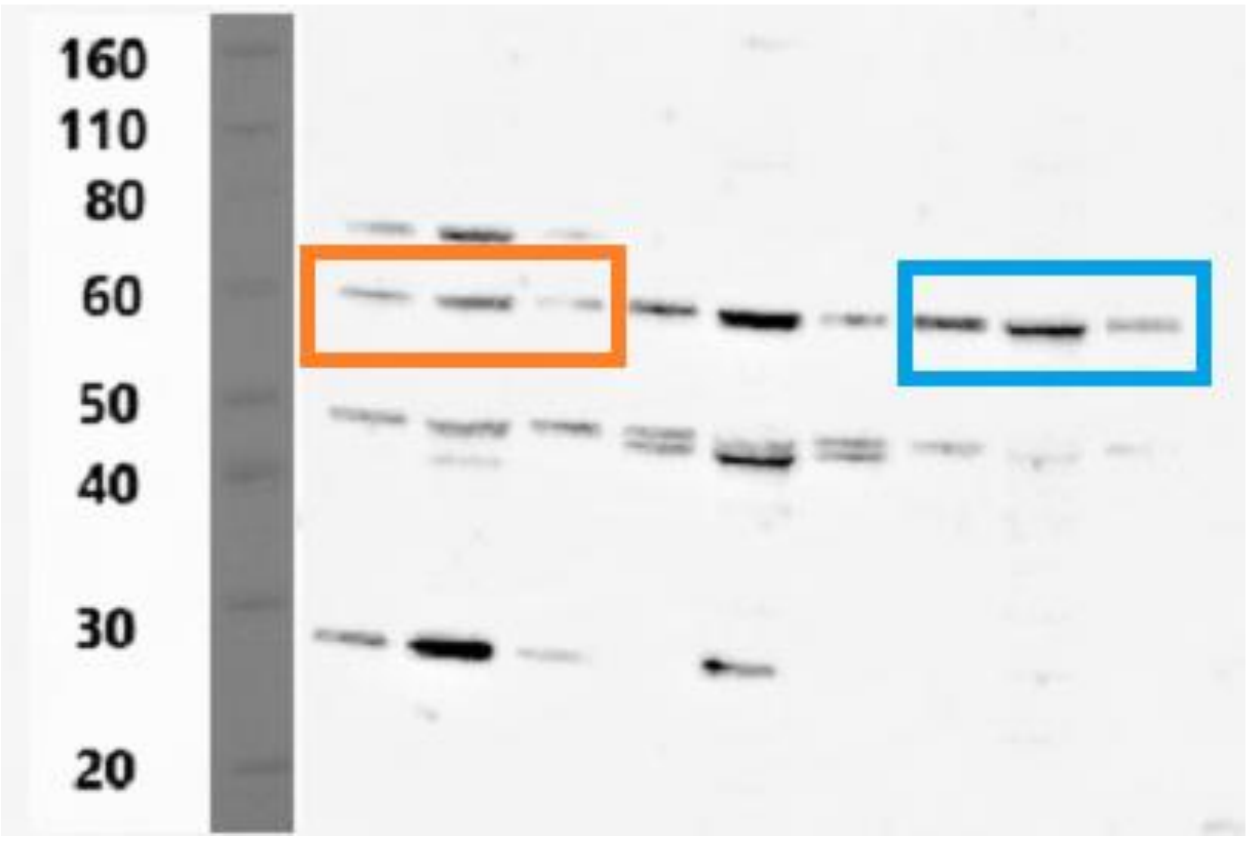

Supplementary Fig. S23. This shows the blotting of p-AKT (Thr308) in OVTOKO (orange) and TOV-112D (blue) in Supplementary Figure S3.

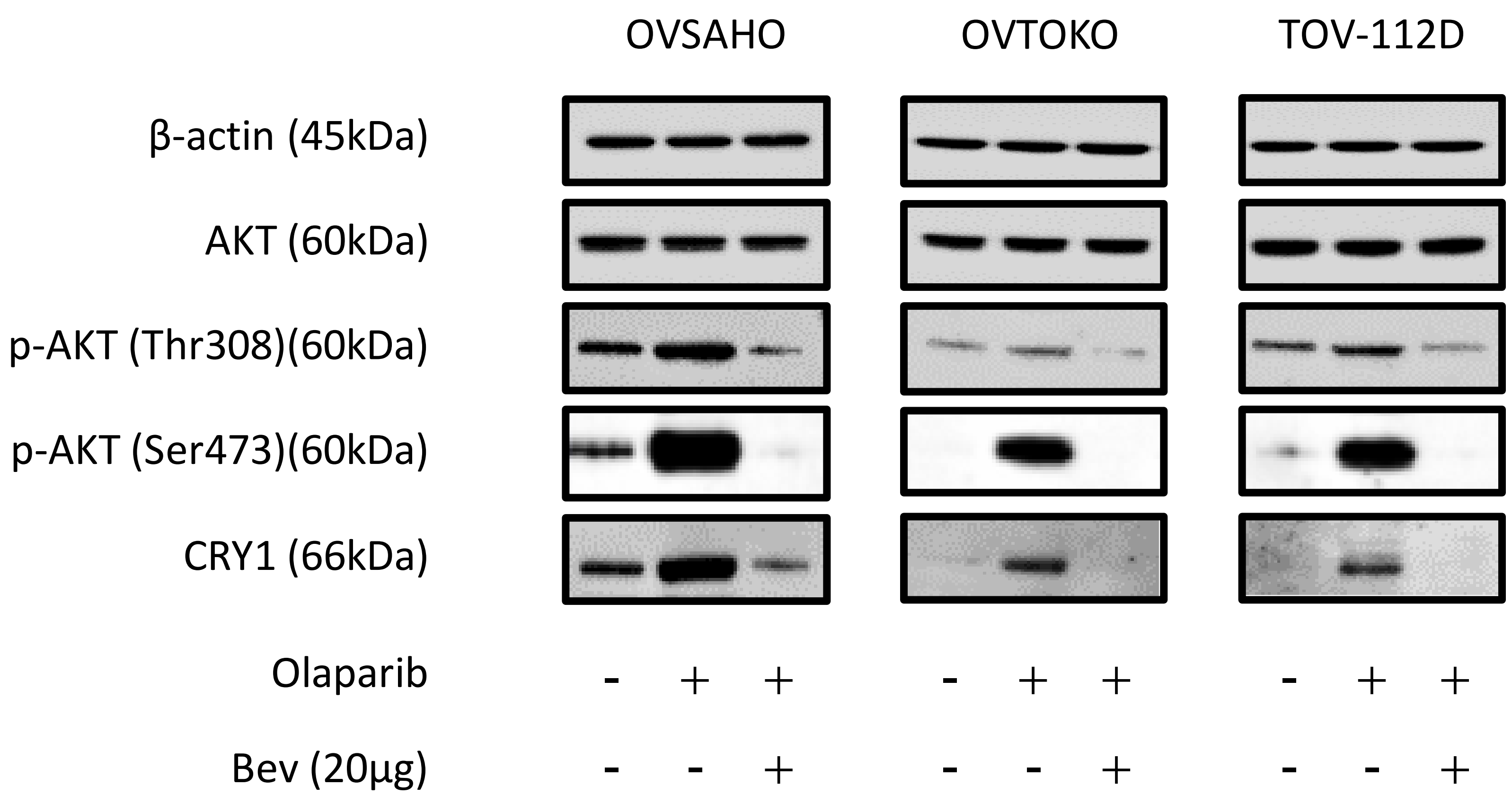

Supplementary Fig. S3.

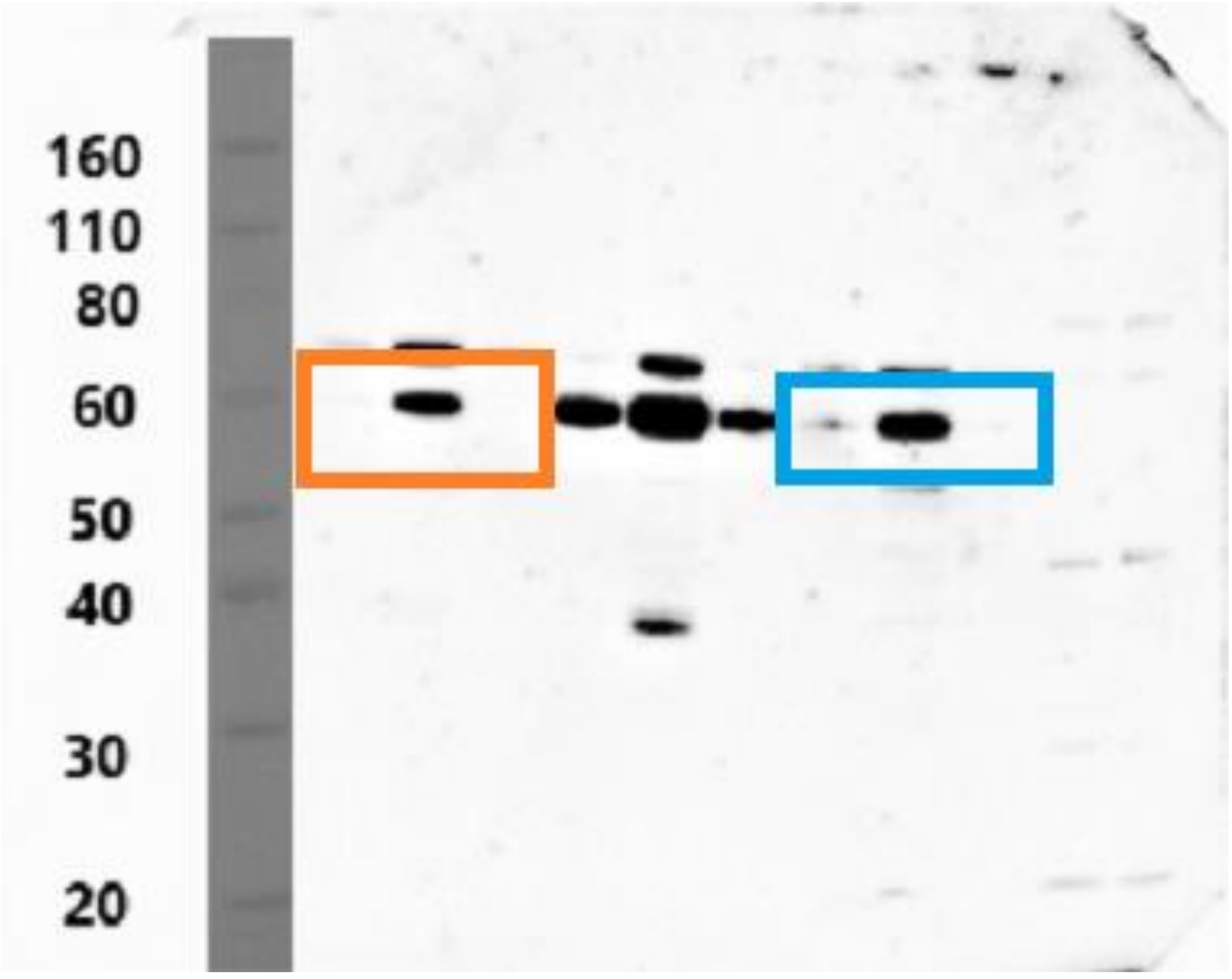

Supplementary Fig. S24. This shows the blotting of p-AKT (Ser473) in OVTOKO (orange) and TOV-112D (blue) in Supplementary Figure S3.

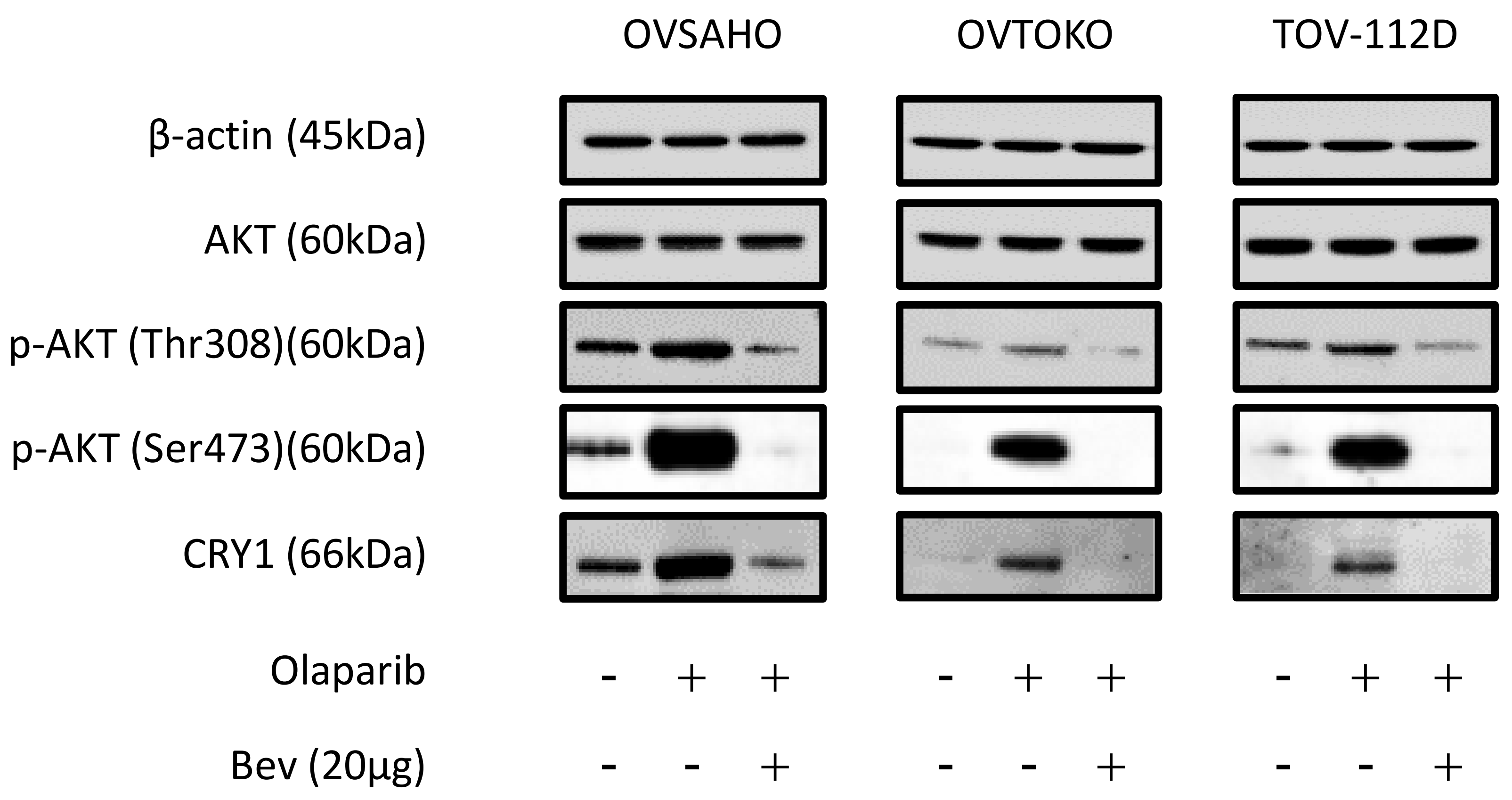

Supplementary Fig. S3.

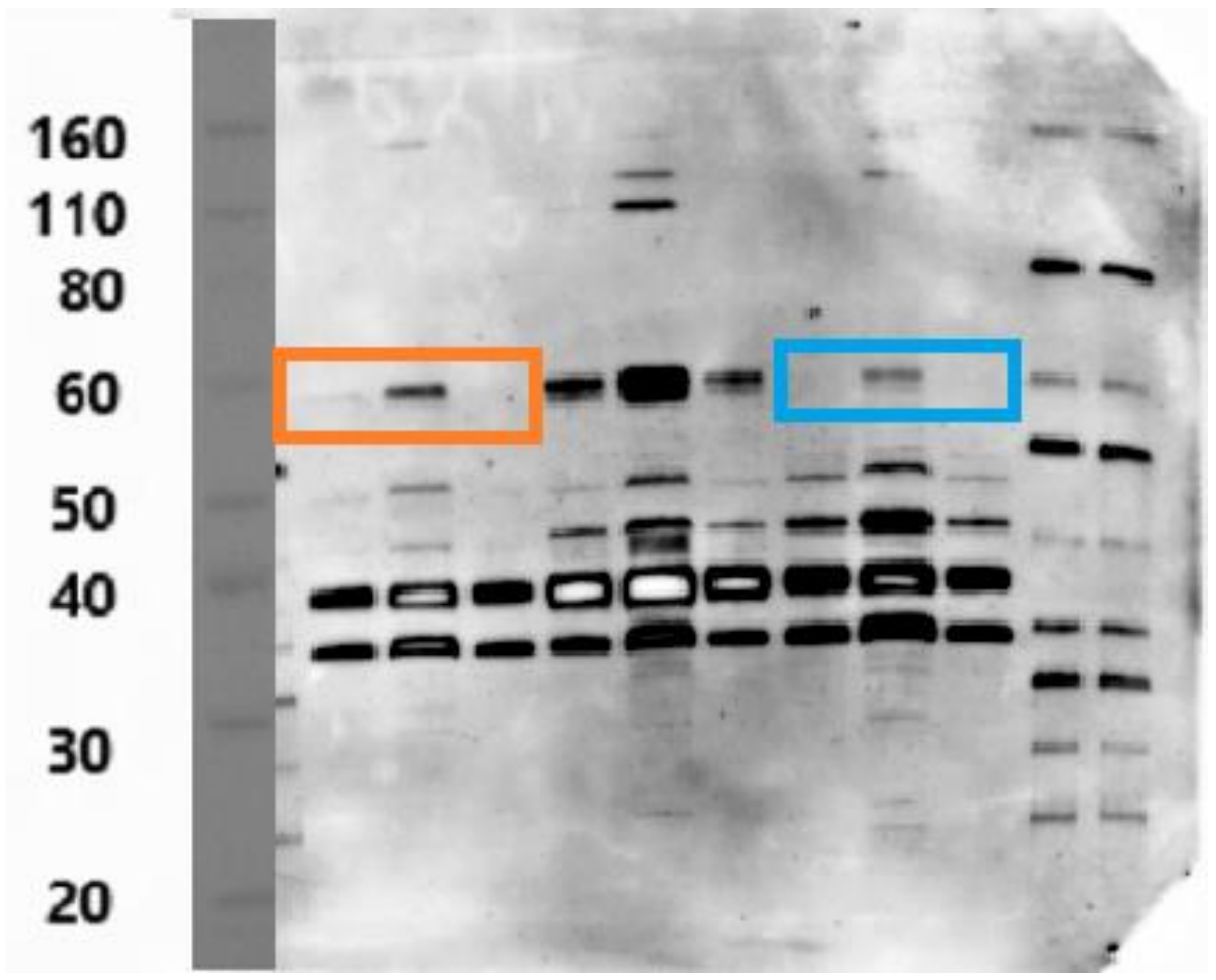

Supplementary Fig. S25. This shows the blotting of CRY1 in OVTOKO (orange) and TOV-112D (blue) in Supplementary Figure S3.
